# Comparative effects of pilates-based interventions on functional mobility, balance, fatigue, and quality of life in people with multiple sclerosis: a systematic review and network meta-analysis

**DOI:** 10.1186/s13102-026-01827-1

**Published:** 2026-07-04

**Authors:** Yehia Nabil, Anas Mansour, Ahmed Adel Mohamed, Kholoud Elsamman, Nourhan Hatem, Yasmin Negida, Aya Samy Salah, Elsayed S. Moubarak, Ragda Mamoun MohamedAhmed Mamoun, Abdelrahman Shata, Salma Allam, Mostafa Meshref

**Affiliations:** 1 Faculty of Medicine, Zagazig University, Zagazig, Egypt; 2 Faculty of Medicine, Al-Azhar University, Cairo, Egypt; 3 Biochemistry and Nutrition Department, Faculty of Women for Science, Ain Shams University, Cairo, 11566 Egypt; 4 Faculty of Medicine, Suez Canal University, Suez, Egypt; 5 Faculty of Medicine, Cairo University, Cairo, Egypt; 6 Faculty of Medicine, Horus University-Egypt, New Damietta, Egypt; 7 Faculty of Medicine, Galala University, Suez, Egypt; 8 Department of Neurology, Faculty of Medicine, Al-Azhar University, Cairo, Egypt

**Keywords:** Multiple Sclerosis, Pilates, Balance, Fatigue, Functional Mobility, Rehabilitation

## Abstract

**Purpose:**

Pilates-based exercise has gained attention as a non-pharmacological rehabilitation strategy for people with multiple sclerosis (MS), yet the comparative effectiveness of different Pilates modalities remains uncertain. This systematic review and network meta-analysis evaluated and ranked Pilates-based interventions for functional mobility, balance, walking performance, fatigue, and health-related quality of life in adults with MS.

**Methods:**

PubMed, Scopus, Web of Science, and Cochrane Central Register of Controlled Trials were searched from inception to October 24, 2025. Eligible studies were randomized controlled trials of structured Pilates-based interventions lasting at least four weeks in adults with MS. The primary outcome was functional mobility measured by the Timed Up and Go test. Secondary outcomes included balance, walking endurance, fatigue, walking performance, and MS-specific quality of life. A frequentist random-effects network meta-analysis was performed; treatment ranking was summarized using SUCRA, risk of bias using RoB 2, and certainty of evidence using GRADE-NMA through CINeMA.

**Results:**

Twenty-two randomized controlled trials involving 901 participants were included. Pilates ranked first for functional mobility and significantly improved Timed Up and Go performance versus control (MD: -5.23 s; 95% CI: -6.39 to -4.06). It also ranked first for balance, with a mean improvement of 8.58 points on the Berg Balance Scale (95% CI: 7.86 to 9.30). Pilates combined with Pilates-based telerehabilitation ranked highest for the 6-Minute Walk Test (MD: 50.81 m; 95% CI: 0.16 to 101.46) and significantly improved Fatigue Severity Scale scores. Home-based Pilates showed the largest reduction in Modified Fatigue Impact Scale scores. No comparison reached high certainty under GRADE-NMA.

**Conclusion:**

Pilates-based interventions may improve mobility, balance, walking endurance, fatigue, and quality of life in people with MS. Sparse networks, risk of bias, heterogeneity, imprecision, and limited follow-up warrant cautious interpretation. Adequately powered trials with standardized protocols are needed before routine implementation can be recommended.

**Graphical Abstract:**

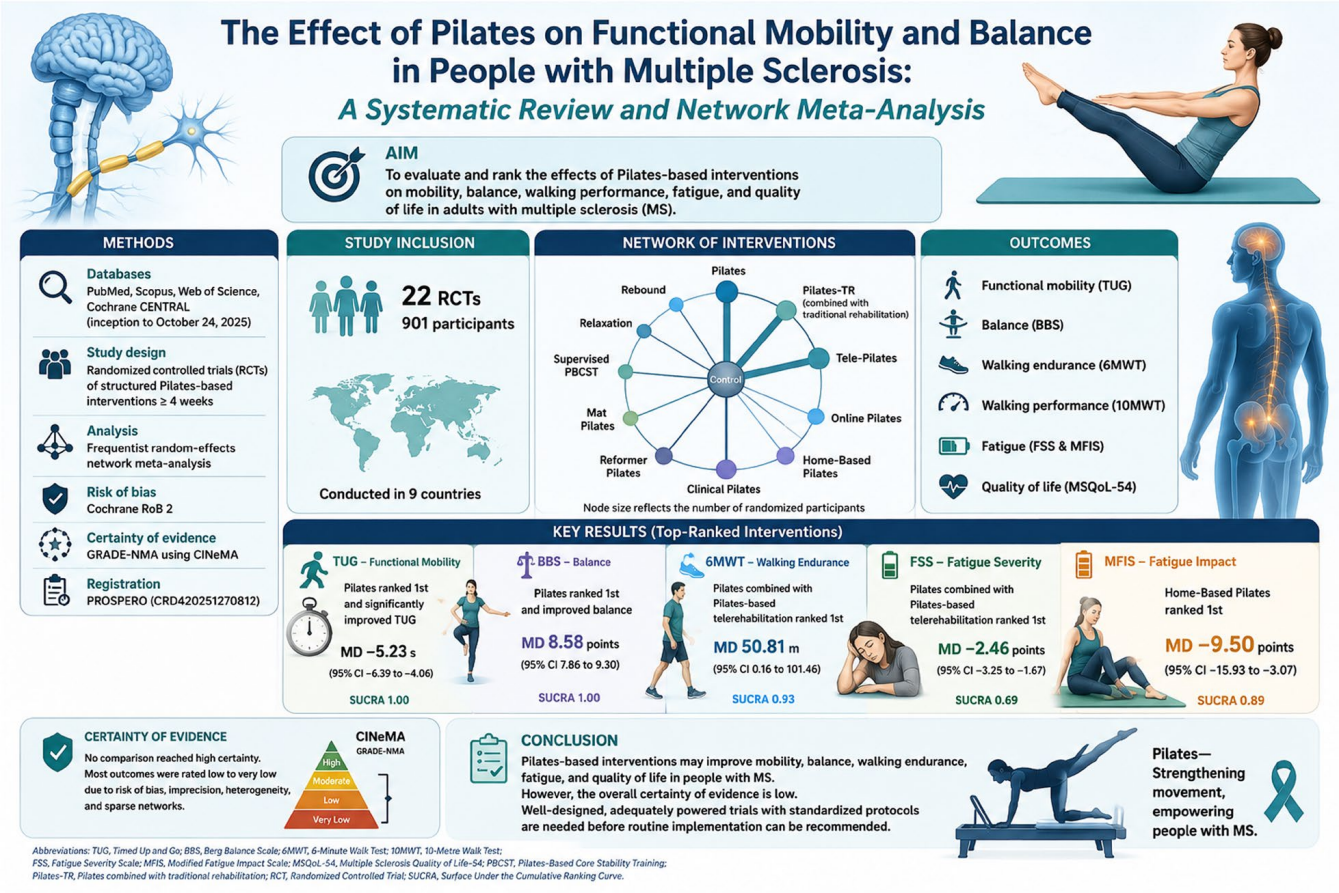

**Supplementary Information:**

The online version contains supplementary material available at 10.1186/s13102-026-01827-1.

## Introduction

Multiple sclerosis (MS) is a chronic, immune-mediated inflammatory disease of the central nervous system [1]. It constitutes the predominant non-traumatic etiology of neurological impairment in young adults [2]. The pathogenic process includes demyelination and neurodegeneration, manifesting as widespread and focal regions of inflammation, gliosis, and neuronal damage in the optic nerve, brain, and spinal cord. Among the most prevalent and debilitating manifestations are impairments in functional mobility, balance, and postural control, which substantially increase the risk of falls and loss of independence [3–5]. These motor deficits, often accompanied by substantial fatigue, significantly impair patient autonomy and quality of life. Consequently, evidence-based rehabilitation interventions constitute a cornerstone of multiple sclerosis management, aiming to preserve functional capacity and promote long-term independence [6].

In recent years, increasing attention has been directed toward effective non-pharmacological management strategies, including mind–body exercise interventions [7]. Pilates is a structured mind–body exercise method that has gained increasing popularity in the management of chronic pain conditions and neurological disorders [8]. It emphasizes awareness of the mind–body connection and the cultivation of conscious motor control, thereby enhancing core stability, postural alignment, physical fitness, neuromuscular coordination, and movement efficiency [9, 10]. The focused and controlled nature of Pilates may be particularly beneficial for individuals with MS, as its emphasis on attention, precision, and regulated movement may mitigate MS-related deficits in proprioception and sensorimotor integration. This capacity to enhance proprioceptive awareness, promote dynamic balance, and restore efficient movement patterns supports its proposed role in optimizing functional outcomes [11].

Recent research has increasingly examined the role of Pilates in managing symptoms associated with multiple sclerosis [12]. Several clinical trials have evaluated its effects on key outcomes such as balance, motor function, and fatigue in individuals with MS [13–15]. However, findings across these studies remain inconsistent and fragmented, limiting the ability to draw definitive conclusions regarding the efficacy of Pilates interventions. Consequently, a conventional pairwise meta-analysis is insufficient to address these complexities. A systematic review incorporating network meta-analysis is therefore warranted to integrate both direct and indirect evidence, enabling comprehensive comparison and ranking of different Pilates-based interventions across various control conditions. Such an approach may offer a more refined understanding of the relative effectiveness of Pilates and support the identification of optimal rehabilitation strategies for individuals with MS.

## Methods

This meta-analysis was conducted in accordance with the Preferred Reporting Items for Systematic Reviews and Meta-Analyses (PRISMA) guidelines and the Cochrane Handbook for Systematic Reviews of Interventions [16, 17]. The review protocol was registered in the International Prospective Register of Systematic Reviews (PROSPERO; registration number: CRD420251270812).

Protocol deviations included restricting the primary analysis to randomized controlled trials, excluding non-randomized controlled studies from the main synthesis, classifying the 2-Minute Walk Test and 10-Meter Walk Test as exploratory walking-performance outcomes, and adding GRADE-NMA/CINeMA certainty assessment during revision in response to peer-review recommendations.

### Search strategy and data sources

PubMed, Scopus, Web of Science, and the Cochrane Central Register of Controlled Trials (CENTRAL) were searched from inception to October 24, 2025. The strategy combined MeSH terms and free-text keywords: (“Multiple Sclerosis” [MeSH] OR “Multiple Sclerosis” OR “MS”) AND (“Pilates” [MeSH] OR “Pilates” OR “Pilates Training” OR “Pilates Exercise”). The PubMed strategy was adapted for other databases and is provided in Table S1. No language or date restrictions were applied. Reference lists of included studies and relevant systematic reviews were manually screened for additional eligible trials.

Records were imported into EndNote for deduplication and then uploaded to Rayyan for title, abstract, and full-text screening.

### Selection criteria

Eligibility was defined using the PICOS framework. The eligible population included adults aged 18 years or older with a clinically definite diagnosis of multiple sclerosis of any subtype; studies enrolling mixed neurological populations were eligible only when MS-specific data were reported separately.

Eligible interventions included any structured exercise program explicitly described as “Pilates,” regardless of modality—mat, reformer, clinical, suspension-based, tele-Pilates, or Pilates combined with Pilates-based telerehabilitation—with a minimum duration of four weeks. Comparators included usual care, waitlist control, no intervention, relaxation, active control, Pilates-based telerehabilitation, physiotherapy, yoga, aquatic exercise, or other non-Pilates programs.

The primary outcome was functional mobility assessed by the Timed Up and Go test. Secondary outcomes were balance (Berg Balance Scale), walking endurance (6-Minute Walk Test), fatigue (Fatigue Severity Scale or Modified Fatigue Impact Scale), and health-related quality of life (MSQOL-54 physical and mental health composites). The 2-Minute Walk Test and 10-Meter Walk Test were analyzed as exploratory walking-performance outcomes where sufficient data were available.

Only randomized controlled trials were eligible. Non-randomized designs—including cohort studies, case-control studies, cross-sectional studies, case reports, case series, protocols without outcome data, narrative reviews, systematic reviews, and conference abstracts without sufficient extractable data—were excluded, as were studies not reporting outcomes of interest. Non-randomized studies identified at full-text screening were recorded in the excluded-studies table with the reason for exclusion.

### Study selection and data extraction

All retrieved records were imported into Rayyan software [18], and duplicate entries were removed. Two independent reviewers screened titles and abstracts against the eligibility criteria; potentially relevant records were retrieved for full-text assessment. Disagreements were resolved by discussion and consensus, or by a third reviewer when necessary.

Data were extracted independently using a standardized, pre-piloted form covering: study characteristics (first author, year, country, design); participant characteristics (sample size, age, sex, MS phenotype, disease duration, EDSS score); intervention characteristics (modality, delivery mode, session duration and frequency, total duration, supervision, co-interventions); comparator characteristics; follow-up duration; and outcome data. For continuous outcomes, extracted data included sample size, baseline and post-intervention values, change scores, standard deviations, standard errors, confidence intervals, or *p*-values. Corresponding authors were contacted when required data were missing or unclear.

Potentially overlapping reports were cross-checked by author group, recruitment setting, sample size, intervention arms, baseline characteristics, follow-up duration, and reported outcomes. Where reports appeared to describe the same or partially overlapping cohort, participants were not double-counted within the same outcome network; the most complete dataset was retained for each outcome and duplicate or overlapping data excluded from the corresponding analysis.

### Risk of bias assessment

The methodological quality and risk of bias of the included studies were independently assessed by three reviewers. Randomized controlled trials were evaluated using the revised Cochrane Risk of Bias tool (RoB 2.0) [19], which assesses bias across five domains: (1) randomization process, (2) deviations from intended interventions, (3) missing outcome data, (4) measurement of outcomes, and (5) selection of the reported result. Each domain was judged as low risk, some concerns, or high risk of bias.

### Data synthesis and statistical analysis

All outcomes were continuous. Effect estimates were expressed as mean differences with 95% confidence intervals, given that outcome measures used identical scales across studies. Positive mean differences indicated favorable effects for outcomes where higher scores reflect improvement (Berg Balance Scale, 6-Minute Walk Test, MSQOL-54); negative mean differences indicated favorable effects for outcomes where lower scores reflect improvement (Timed Up and Go, Fatigue Severity Scale, Modified Fatigue Impact Scale, timed walking tests).

When change-score standard deviations were unavailable, they were derived from confidence intervals, standard errors, *p*-values, or baseline and final standard deviations per Cochrane Handbook methods, or imputed using an assumed correlation of 0.5. Sensitivity analyses were planned to examine the effect of imputed variance data when sufficient studies were available.

A frequentist network meta-analysis framework was used to synthesize direct and indirect evidence across multiple interventions simultaneously. A random-effects model was prespecified given anticipated clinical and methodological heterogeneity across trials. For each outcome, network plots depicted the evidence geometry: nodes represented interventions or comparators (sized by participant count), and edges represented direct comparisons (weighted by study count). Network connectivity was examined before synthesis.

Within-comparison heterogeneity was assessed using I², with values of approximately 25%, 50%, and 75% interpreted as low, moderate, and high. Overall network heterogeneity was estimated from the total I² of each network model.

Consistency was assessed where network geometry permitted. Local inconsistency was evaluated using side-splitting comparisons and global inconsistency using the design-by-treatment interaction model, where closed loops and sufficient direct and indirect evidence existed. For star-shaped or open networks without closed loops, formal testing was not feasible; these outcomes were interpreted qualitatively based on network geometry, transitivity, confidence intervals, and certainty of evidence.

The transitivity assumption was evaluated by comparing potential effect modifiers across treatment comparisons, including age, sex distribution, MS phenotype, baseline disability, intervention duration, supervision, delivery mode, and comparator type. Network estimates were interpreted cautiously where substantial imbalance existed.

Intervention ranking was summarized using ranking probabilities and the Surface Under the Cumulative Ranking Curve (SUCRA), with values from 0% to 100% (higher = greater probability of being among the most effective). Rankings were treated as descriptive and supplementary, interpreted alongside effect estimates, confidence intervals, certainty of evidence, risk of bias, network geometry, and clinical relevance.

Results were presented as forest plots, network plots, league tables, rankograms, cumulative ranking plots, and summary-of-findings tables. League tables reported pooled mean differences with 95% confidence intervals for all pairwise comparisons.

Subgroup and sensitivity analyses were planned a priori and conducted only where sufficient studies remained within each comparison after stratification or exclusion of high-risk-of-bias studies. Where the number of studies per comparison was too small, these analyses were not feasible and are reported as such.

All analyses used R version 4.3.0. The netmeta package was used for network meta-analysis, inconsistency assessment, treatment ranking, and contribution matrix generation; igraph was used for network visualizations.

### Certainty of evidence using GRADE for network meta-analysis

Certainty of evidence was assessed using the GRADE approach for network meta-analysis, operationalized through the Confidence in Network Meta-Analysis (CINeMA) framework at the network-estimate level [20, 21]. The principal comparison for each outcome in the summary-of-findings table was the highest-ranked intervention by SUCRA versus the common control reference. Two reviewers independently conducted the certainty assessment; disagreements were resolved by discussion or third-reviewer adjudication.

Because the analysis was restricted to randomized controlled trials, each network estimate began at high certainty and was rated down when important concerns were identified across six domains: within-study bias, reporting bias, indirectness, imprecision, heterogeneity, and incoherence.

Within-study bias was informed by RoB 2 judgments interpreted relative to each study’s proportional contribution to the relevant network estimate. Reporting bias was assessed through comparison-adjusted funnel plots where feasible, small-study effects, publication status, selective outcome reporting, and the number of contributing studies. Indirectness was judged on the applicability of the population, intervention, comparator, and outcome, supported by the contribution matrix.

Imprecision was evaluated by examining whether the 95% confidence interval crossed the line of no effect or spanned both clinically unimportant and important effects, with additional weight given to limited information size in sparse networks. Heterogeneity was rated according to whether between-study variability could plausibly alter the clinical interpretation of the estimate. Incoherence was evaluated using direct-versus-indirect evidence in closed loops where network geometry permitted formal testing; in star-shaped or open networks without closed loops, it was treated as statistically non-estimable and interpreted qualitatively against transitivity and clinical plausibility.

Final certainty ratings were classified as high, moderate, low, or very low. The GRADE summary-of-findings table reports the network mean difference, 95% confidence interval, participant and study counts, certainty rating, and explicit downgrading rationale for each outcome. SUCRA values and rank probabilities served only to identify the summary comparison and were not used to upgrade or determine certainty ratings.

## Results

### Study selection

**Fig. 1:**
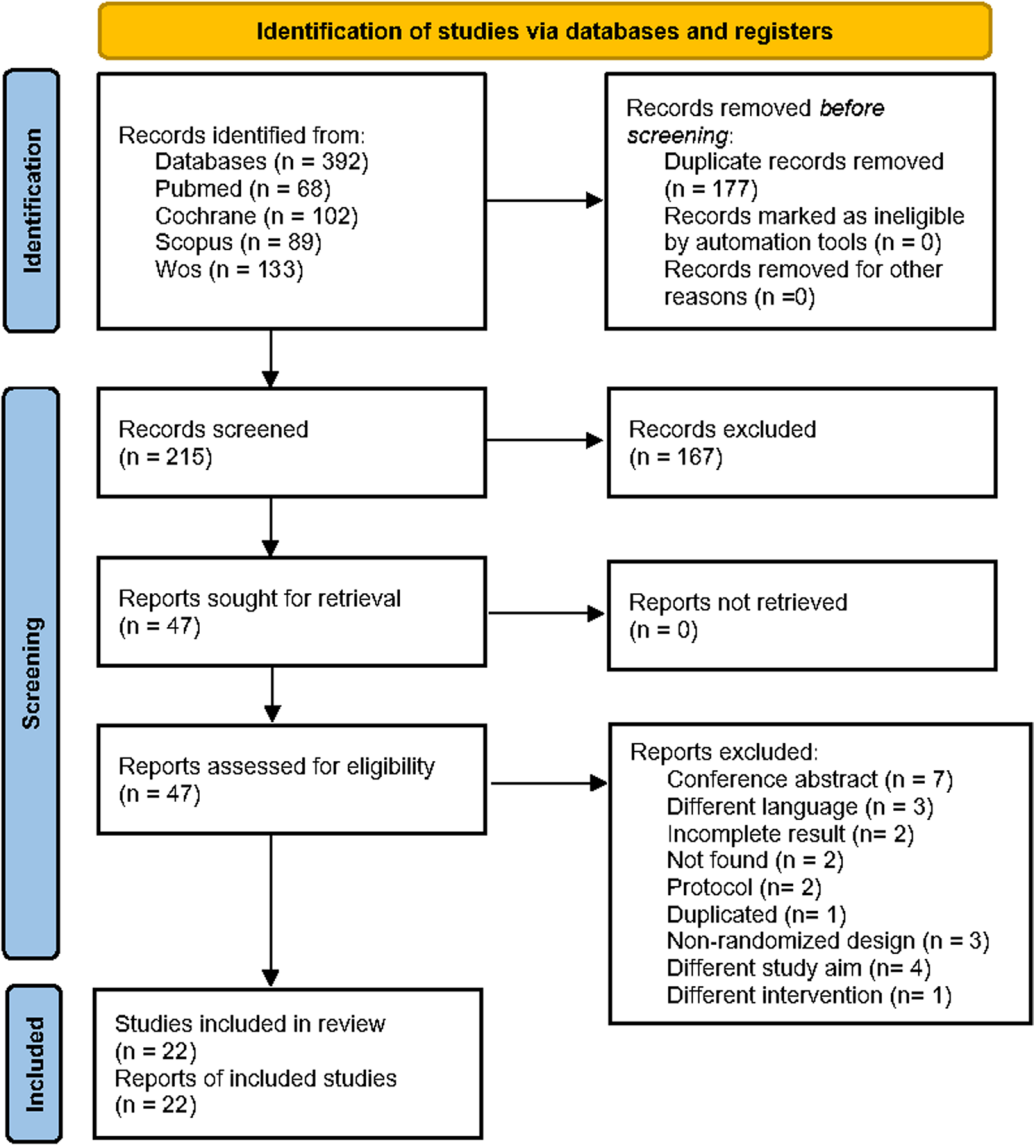
PRISMA Flowchart

Database searches across PubMed, CENTRAL, Scopus, and Web of Science yielded 392 records. The complete database-specific search strategies are provided in Supplementary Table S1. Removing 177 duplicates left 215 records for title and abstract screening, of which 168 were excluded for failing to meet eligibility criteria. The remaining 47 full-text reports underwent detailed assessment; 25 were subsequently excluded — due to conference abstracts lacking extractable data, non-English texts without usable data, unavailable full texts, protocols without outcome data, duplicate reports, non-randomized designs, divergent study aims, or ineligible interventions. Twenty-two randomized controlled trials were included in the systematic review and network meta-analysis. The selection process is illustrated in Fig. 1.

### Study characteristics

Twenty-two randomized clinical trials conducted across six countries -- Turkey, Iran, Ireland, Canada, the United Kingdom, and Israel -- were included. Published between 2013 and 2025, the trials collectively enrolled 901 participants with multiple sclerosis, and their key characteristics are summarized in Table 1. Individual sample sizes ranged from 20 to 100 participants, averaging approximately 41 per study. Reported group mean ages ranged from roughly 30 to 55 years. Most study groups were predominantly female, though some trials enrolled only male participants or did not clearly report sex distribution. Where specified, relapsing-remitting multiple sclerosis was the most common disease phenotype; one study also included secondary progressive MS. Eligibility criteria generally targeted mild-to-moderate disability, with EDSS thresholds ranging from 0 to 6.5 and reported group mean EDSS values from approximately 1.2 to 5.0, as detailed in Table 2.

**Table 1:** Summary of included studies

Study ID	Study Design	Country	No. of Participants	Population	Intervention	Comparator	Outcomes	Key Finding
Eldemir 2025 [22]	Blind RCT	Turkey	28	PwMS, age 18–65 yrs, EDSS 0–5, w/o MS attack in the last 3 mo.	Online PG	CG	Cognitive Functions: MoCA + TMT-A & TMT-B + ST-A, ST-B & ST-C. DT Performance: postural stability during mental tracking & verbal fluency, walking parameters during cognitive tasks, functional mobility also during cognitive tasks.	Online PG significantly improved cognitive, psychomotor, & gait measures.
Estiri 2025 [23]	RCT	Iran	29	F PwMS, age 20–50 years, EDSS 2–5, MS duration ~ 6–7 yrs.	1. PG w/o Music 2. PG With Music	CG	Static balance, body oscillations, dynamic Balance assessed, QoL.	PG—whether with or w/o music—significantly improved static balance, dynamic balance, & QoL, though outcomes were slightly better with PG w/o music.
Ghasemi 2025 [24]	Single-blind RCT	Iran	22	F PwMS, EDSS 2–6, MS duration ~ 17–18 yrs, w/o MS attack in the last 30 days.	1. PSBR 2. PSJR	CG	Fear of falling, QoL, MMSE, FFMQ.	Both PSBR & PSJR—paired with either Benson or Jacobson relaxation—significantly reduced fear of falling & improved QoL, w/o meaningful difference between the 2 relaxation approaches.
Eldemir 2024 [14]	Blind RCT	Turkey	30	PwMS, age 18–65 yrs, EDSS 0–5 w/o MS attack in the last 3 mo.	PG-TR	CG	Extremity muscle strength, core endurance, core power, balance, spatio-temporal gait analysis, functional exercise capacity, fatigue, QoL.	PG-TR improved extremity strength, core endurance & power, balance, walking speed & cadence, functional exercise capacity, fatigue, & QoL.
Ghasemi 2024 [25]	Single-blind RCT	Iran	22	F PwMS, mean age 44.27 ± 7.35 yrs, EDSS 2–6.5.	1. PSBR 2. PSJR	CG	DGI, MCQ-30, MMSE.	Both PSBR & PSJR combined with self-awareness—whether mental or physical—significantly improved gait & metacognitive ability.
Miri 2023 [26]	RCT	Iran	40	F PwMS, mean age 25–50 yrs, EDSS < 5.5.	PG	CG	Balance, fatigue, muscular endurance (core & general), QoL.	PG significantly improved balance, fatigue, & QoL, but produced limited gains in core muscle endurance.
Najafi 2023a [27]	RCT	Iran	82	PwMS, mean age 41.51 ± 7.06 yrs, EDSS 0–6.5, w/o exercise for or corticosteroids in last 3 mo.	1. Tele-PG 2. Tele-Yoga	CG	QoL, depression, mental health, physical activity level, physical & mental health composites, pain, energy, emotional well-being, role limitations (physical & emotional), social function, cognitive function, health perception, sexual function.	Tele-yoga & tele-PG improve physical & mental health & QoL in both RRMS & SPMS patients, with comparable benefits across phenotypes.
Najafi 2023b [28]	RCT	Iran	45	F with RRMS, age 18–65 yrs, EDSS 0-5.5, BMI 20–32 kg/m².	1. Tele-PG 2. Tele-Yoga	CG	Prolactin & cortisol serum levels, depression, mental health, physical activity level, walking speed, QoL.	Both tele-yoga & tele-PG produced significant increases in prolactin, reductions in cortisol, & improvements in depression, physical activity, walking speed, & overall QoL, while mental health scores improved without a significant difference between groups.
Fleming 2021 [29]	Single-blind RCT	Ireland	80	PwMS, age > 18 yrs, PDDS < 3, w/o previous Pilates experience.	Home-based PG	WLc	Anxiety, depressive, MFIS assessed (physical, cognitive, psychosocial, total fatigue).	Home-based PG produced statistically significant improvements across all outcomes, reducing depressive symptoms, anxiety, & cognitive, physical, psychosocial, & total fatigue; results were similar in women-only analyses.
Gheitasi 2021 [30]	RCT	Iran	30	PwMS, age 25–40 yrs, DSI 3–5, w/o MS attack or participating in exercise in the last 2 mo.	PG	CG	BBS, TUG, FRT.	PG significantly improved functional balance scores, w/o adverse or harmful events reported in any group.
Güngör 2021 [31]	Single-blind RCT	Turkey	42	PwMS, age 20–65 yrs, EDSS < 6 w/o MS attack in the last 3 mo.	PBCST at Clinic	Home-based PBCST	Knee muscle strength, postural sway.	Both groups showed significant improvements across outcomes, though the supervised group was generally superior; only select postural-sway subparameters failed to improve in the home-based PBCST group.
Abasıyanık 2020 [32]	RCT	Turkey	33	PwMS, age > 18 yrs, walking 100 m independently,	PG	Home-based exercise training	Balance, walking, fall risk, respiratory, cognitive functions.	PG improved walking endurance, postural & core stability, respiratory function, & cognitive performance, w/o significant differences in walking speed, perceived walking ability, or fear of falling.
Duff 2018 [33]	Single-blind RCT	Canada	30	Ambulatory PwMS, PDDS < 7.	PG & Massage therapy	Massage therapy only	Walking performance, functional ability, balance, flexibility, body composition, core endurance, muscle strength, voluntary activation.	PG improved walking distance & TUG decreased.
Eftekhari 2018a [34]	RCT	Iran	25	F PwMS, EDSS 2–6.	PG	CG	Anthropometric variables (BW, BMI), body circumferences (WC, HC, MAMC, CC), WHR, 7-site skinfold*, FP, FM, FFM, BD, functional indices (balance, walking speed, endurance, fatigue).	PG significantly reduced BW, BMI, WC, HC, MAMC, skinfold measures, FP, FM, & fatigue, while increasing bone density, balance, walking speed, & endurance.
Eftekhari 2018b [34]	RCT	Iran	25	F PwMS (RRMS).	PG	CG	Serum levels of IL-10 & BDNF.	PG had no significant change in IL-10 but significantly increased BDNF.
Bulguroglu 2017 [13]	RCT	Turkey	38	PwMS, mean age ≈ 40 yrs, EDSS ≤ 4.5.	PG	Home-based relaxation & breathing exercises	Balance, functional mobility, core stability, Fatigue, QoL.	Both Mat & Reformer PG significantly improved balance, mobility, core stability, fatigue, & QoL, w/o differences between modalities, although Reformer yielded greater trunk-flexor endurance.
Fox 2016 [35]	Blind RCT	United Kingdom	100	PwMS (mostly RRMS), mean age ≈ 49 yrs.	PBCST	CG	BBS, TUG, 10MWT, MSWS, FSS, MSQOL-54.	PG significantly improved balance & walking ability, & reduced fatigue.
Kalron 2017 [36]	RCT	Israel	45	PwMS (RRMS), age > 18 yrs, EDSS 4.3.	PG	Bobath-based physical therapy	Gait & balance measures, clinical tests (TUG, 2- & 6-MWT, Functional Reach, BBS, Four Square Step), self-reports (MSWS, MFIS).	Both Pilates & PT significantly improved walking speed, step length, & balance; no significant difference between groups — Pilates is as effective as conventional physical therapy for improving walking & balance in MS patients.
Küçük 2016 [37]	RCT	Turkey	20	Ambulatory PwMS, age > 18 yrs, EDSS ≤ 6.	PG	CG	MSFC, BBS, TUG, TIS, MFIS, BDI, MusiQoL.	PG significantly improved cognition, QoL, balance, fatigue, & physical performance, with no major effect on depression, supporting its role as a holistic physiotherapy approach for MS.
Hosseini 2013 [38]	RCT	Iran	45	M PwMS, mean age ≈ 31 yrs, EDSS 0–4.	PG	CG	Static & dynamic balance.	Both PG & rebound therapy significantly improved static & dynamic balance. PG was slightly more effective for static balance, while rebound therapy was better for dynamic balance. No significant difference between the 2 interventions overall.
Marandi 2013a [39]	RCT	Iran	45	F PwMS, age 20–40 yrs, EDSS < 4.5, MS duration ≈ 8 yrs.	PG	CG	Dynamic balance (right & left leg performance).	Both PG & aquatic training significantly improved dynamic balance w/o significant difference between PG & aquatic training.
Marandi 2013b [39]	RCT	Iran	45	F PwMS, age 20–40 yrs, EDSS < 4.5, MS duration ≈ 8 yrs.	PG	CG	Hand grip strength (dominant & non-dominant).	Both PG & aquatic training significantly improved hand grip strength in both hands w/o significant difference between PG & aquatic training.

*ABC* Activities-specific Balance Confidence, *BD* body density, *BDI* Beck Depression Inventory, *BDNF* Brain-Derived Neurotrophic Factor, *BBS* Berg Balance Scale, *BMI* body mass index, *BW* body weight, *CC* calf circumference, *CG *control group, *DGI* Dynamic Gait Index, *DSI* Disability Score Index, *DT *dual-task, *EDSS* Expanded Disability Status Scale, *F* female, *FFM* fat-free mass, *FFMQ* Five Facet Mindfulness Questionnaire, *FM* fat mass, *FP* fat percentage, *FRT* Functional Reach Test, *FSS* Fatigue Severity Scale, *HC* hip circumference, *IL* interleukin, *M* male, *MAMC* mid-arm muscle circumference, *MCQ-30* Metacognition Questionnaire-30, *MFIS* Modified Fatigue Impact Scale, *MMSE* Mini-Mental State Examination, Mo months, *MoCA* Montreal Cognitive Assessment, *MS* multiple sclerosis, *MSFC* Multiple Sclerosis Functional Composite, *MSQOL-54* Multiple Sclerosis Quality of Life–54, *MSWS-12* Multiple Sclerosis Walking Scale–12, *MWT* Minute Walk Test, *NA* not available, *PBCST* Pilates-based Core Stabilization Training, *PDDS* Patient-Determined Disease Steps, *PG* Pilates group, *PG-TR* Pilates-based Telerehabilitation group, *Pilates-TR* Pilates-based Telerehabilitation, *PwMS* patients with multiple sclerosis, *PSBR* Pilates Suspension with Benson Relaxation, *PSJR* Pilates Suspension with Jacobson Relaxation, *QoL* quality of life, *RCT* randomized controlled trial, *RRMS* relapsing–remitting multiple sclerosis, *SD* standard deviation, *SPMS* secondary progressive multiple sclerosis, *ST* Stroop test, *T25-FW* Timed 25-Foot Walk, *TIS* Trunk Impairment Scale, *TMT* Trail Making Test, *TUG* Timed Up & Go, *WC* waist circumference, *WHR* waist-to-hip ratio, *WLc* wait-list control, *w/o* without, *yrs* years

*Chest, abdominal, thigh, triceps, subscapular, suprailiac, & midaxillary

**Table 2:** Baseline Characterisitics of the included studies

Study ID	Group	Sample Size	Age mean (SD) years	Female *n* (%)	Disease Duration in year mean (SD)	Disability score EDSS mean (SD)	Follow-up Duration mean (SD)	Total duration (weeks)	Number of sessions per week	Session length (minutes)	Supervision level
Eldemir 2025 [22]	Online Pilates	14	43.57 (7.79)	12 (85.71%)	12.89 (4.90)	1.78 (0.80)	NA	6	3	60	One-on-one via videoconference with physiotherapist
	Control	14	39.07 (9.92)	12 (85.71%)	11.14 (3.84)	1.78 (0.80)	NA	6	NA	NA	Waitlist, no intervention
Estiri 2025 [23]	Pilates with music	10	39.53 (7.51)	10 (100%)	6.8 (0.1)	3.1 (0.5)	NA	6	3	60	Supervised by Pilates instructor & exercise physiologist
	Pilates without music	10	39.6 (6.83)	10 (100%)	6.2 (1.1)	3.2 (0.1)	NA	6	3	60	Supervised by Pilates instructor & exercise physiologist
	Control	9	38.6 (6.71)	9 (100%)	6.6 (1.4)	3.8 (1.1)	NA	6	NA	NA	No supervised exercise, daily activities only
Ghasemi 2025 [24]	PSBR	7	45.28 (5.4)	7 (100%)	18 (7.7)	5 (1.15)	NA	7	3	60	Remote, online with family member present
	PSJR	8	42.37 (5.92)	8 (100%)	17.25 (5.33)	4.5 (1.85)	NA	7	3	60	Remote, online with family member present
	Control	7	45.42 (10.56)	7 (100%)	18.57 (8.75)	5 (1)	NA	7	0	0	No intervention
Eldemir 2024 [14]	Pilates-TR	15	41.00 (± 7.82)	14 (93.3%)	9.7 (5.7)	1.7 (1.2)	NA	6	3	60	Remote, via videoconference (Zoom/WhatsApp), real-time therapist supervision
	Control	15	38.40 (10.86)	14 (93.3%)	9 (5.7)	1.8 (1.6)	NA	6	0	0	No intervention
Ghasemi 2024 [25]	PSBR	7	45.28 (5.4)	7 (100%)	18 (7.7)	5 (1.15)	NA	7	3	60	Supervised (online with family assistance)
	PSJR	8	42.37 (5.92)	8 (100%)	17.25 (5.33)	4.5 (1.85)	NA	7	3	60	Supervised (online with family assistance)
	Control	7	45.42 (10.56)	7 (100%)	18.57 (8.75)	5 (1)	NA	7	NA	NA	NA
Miri 2023 [26]	Pilates training	20	NA	20 (100%)	10.4 (3.5)	3.6 (1.6)	NA	8	3	60–90 (increased over time)	In-person, physiotherapist & corrective exercise expert
	Control	20	NA	20 (100%)	10.4 (3.5)	3.6 (1.6)	NA	8	NA	NA	NA
Najafi 2023a [27]	Tele-Pilates	29	40.72 (5.93)	23 (79.3%)	NA	3.74 (1.64)	8 weeks	8	3	60	Remote
	Tele-Yoga	26	41.85 (6.94)	21 (80.8%)	NA	4.17 (1.92)	8 weeks	8	3	60	Remote
	Control	27	39.89 (6.03)	21 (77.8%)	NA	3.37 (1.76)	8 weeks	8	NA	NA	NA
Najafi 2023b [28]	Tele-Pilates	15	36.20 (4.33)	15 (100%)	10.93 (4.38)	2.50 (1.32)	8 weeks	8	3	60	Remote
	Tele-Yoga	15	37.40 (6.03)	15 (100%)	8 (5.84)	2.50 (1.19)	8 weeks	8	3	60	Remote
	Control	15	40.40 (5.35)	15 (100%)	9.27 (8.37)	2.66 (1.03)	8 weeks	8	NA	NA	NA
	Clinical Pilates	16	42.50 (6.76)	12 (75%)	12.59 (6.23)	3.06 (1.65)	8 weeks	8	1	60	Supervised group sessions once weekly plus home exercises
Fleming 2021 [29]	Home-based Pilates	39	47.1 (10.0)	36 (92.3%)	NA	NA	8 weeks	8	2	NA	Home-based (DVD-guided) with weekly phone support
	Wait-List Control	41	47.4 (10.2)	33 (80.5%)	NA	NA	8 weeks	8	2	NA	NA
Gheitasi 2021 [30]	Pilates training	15	30.60 (5.27)	0 (0%)	5.50 (1.5)	4.60 (1.60)	12 weeks	12	3	60	Supervised (clinic-based)
	Control	15	32.10 (6.30)	0 (0%)	4.00 (1)	4.50 (1.1)	12 weeks	NA	NA	NA	NA
Güngör 2021 [31]	Supervised PBCST	22	41.2 (9.9)	20 (91%)	7.4 (6.1)	3.03 (1.25)	NA	8	2	60–75	Supervised by physiotherapist in clinic
	Home-based PBCST	20	37.5 (11.9)	16 (80%)	7.9 (5.4)	2.95 (1.29)	NA	8	2	60–75	Home-based with bi-weekly clinic visits and weekly phone follow-up
Abasıyanık 2020 [32]	Clinical Pilates	16	42.50 (6.76)	12 (75%)	12.59 (6.23)	3.06 (1.65)	NA	8	1	55–60	Supervised (by physiotherapist)
	Home Exercise	17	48.24 (11.79)	11 (64.7%)	9.83 (8.7)	3.24 (1.77)	NA	8	3	NA	Unsupervised (Monitored by telephone)
	Control	15	33.9 (6)	15 (100%)	NA	NA	NA	8	0	NA	NA
Duff 2018 [33]	Pilates	15	45.7 (9.4)	12 (80%)	NA	NA	NA	12	3	50	Certified Pilates instructors
	Control	15	45.1 (7.4)	11 (73%)	NA	NA	NA	12	1	60	NA
Eftekhari 2018a [34]	Pilates	13	34.46 (7.29)	13 (100%)	NA	NA	NA	8 weeks	3	50–60	Supervised (Clinical)
	Control	12	31.41 (8.89)	12 (100%)	NA	NA	NA	8 weeks	NA	NA	NA
Eftekhari 2018b [34]	Pilates	13	34.46 (7.29)	13 (100%)	NA	NA	NA	8	3	40–50	Supervised (Implied by center setting and instruction)
	Control	12	31.41 (8.89)	12 (100%)	NA	NA	NA	8	NA	NA	NA
Bulguroglu 2017 [13]	Mat Pilates	12	44.6 (8.5)	NA	6.9 (8.6)	2 (1.8)	NA	8	2	60–90	Supervised (Physiotherapist/Pilates trainer)
	Reformer Pilates	13	35.5 (8.7)	NA	5.7 (6.6)	2 (1.7)	NA	8	2	60–90	Supervised (Physiotherapist/Pilates trainer)
	Control	13	36.3 (14.1)	NA	4.2 (6.2)	1.2 (1.2)	NA	8	2	NA	Unsupervised (Home program)
Fox 2016 [35]	Pilates	33	53.97 (9.19)	28 (84.9%)	18.94 (11.29)	NA	4 weeks	12	1	30	Individualized, one-to-one
	Standardized Exercises	35	54.60 (11.54)	25 (71.4%)	18.46 (11.59)	NA	4 weeks	12	1	30	Individualized, one-to-one
	Relaxation	32	53.78 (9.72)	21 (65.6%)	20.53 (10.96)	NA	4 weeks	12	0.25	60	Individualized face-to-face
Kalron 2017 [36]	Pilates	22	42.9 (7.2)	15 (68.2%)	11.3 (6.9)	4.1 (1.1)	NA	12	1	30	Individualized face-to-face
	Standardized Physical Therapy	23	44.3 (6.6)	14 (60.9%)	12.4 (5.7)	4.6 (1.3)	NA	12	1	30	Individualized face-to-face
Küçük 2016 [37]	Pilates	11	47.2 (9.5)	7 (63.6%)	14.8 (7.4)	3.2 (2.2)	NA	8	2	45–60	Supervised by a physical therapist
	Control	9	49.7 (8.9)	6 (66.7%)	14.2 (9.5)	2.8 (1.4)	NA	8	2	NA	NA
	Control	8	36.3 (15.6)	8 (100%)	2.06 (2.7)	1.96 (1.9)	NA	8	2	NA	Home exercise (monitored via phone)
Hosseini 2013 [38]	Rebound therapy	15	32.21 (7.6)	0	NA	NA	NA	8	3	30	NA
	Pilates	15	30.32 (8.32)	0	NA	NA	NA	8	3	30	NA
	Control	15	31.43 (7.09)	0	NA	NA	NA	NA	NA	NA	NA
Marandi 2013a [39]	Pilates	15	NA	15 (100%)	8 (2)	NA	NA	12	3	60	Supervised
	Aquatic training	15	NA	15 (100%)	8 (2)	NA	NA	12	3	60	Supervised
	Control	15	NA	15 (100%)	8 (2)	NA	NA	12	NA	NA	NA
Marandi 2013b [39]	Pilates	15	NA	15 (100%)	8 (2)	NA	NA	12	3	60	NA
	Aquatic training	15	NA	15 (100%)	8 (2)	NA	NA	12	3	60	NA
	Control	15	NA	15 (100%)	8 (2)	NA	NA	12	NA	NA	NA

*Abbreviations*: *NA* Not Applicable, *PSBR* Pilates Suspension training with Benson Relaxation, *PSJR* Pilates Suspension training with Jacobson Relaxation, *Pilates-TR* Pilates-based Telerehabilitation, *PBCST* Pilates-Based Core Stability Training

Outcome assessments spanned balance, functional mobility, walking performance, fatigue, quality of life, cognition, psychological outcomes, and selected biological or anthropometric measures. Commonly reported instruments included the Berg Balance Scale, Timed Up and Go test, the 2-Minute, 6-Minute, and 10-Meter Walk Tests, and fatigue scales including the Fatigue Severity Scale and Modified Fatigue Impact Scale. Only outcomes reported sufficiently across studies were carried into the network meta-analysis.

### Risk of bias of included studies

**Fig. 2:**
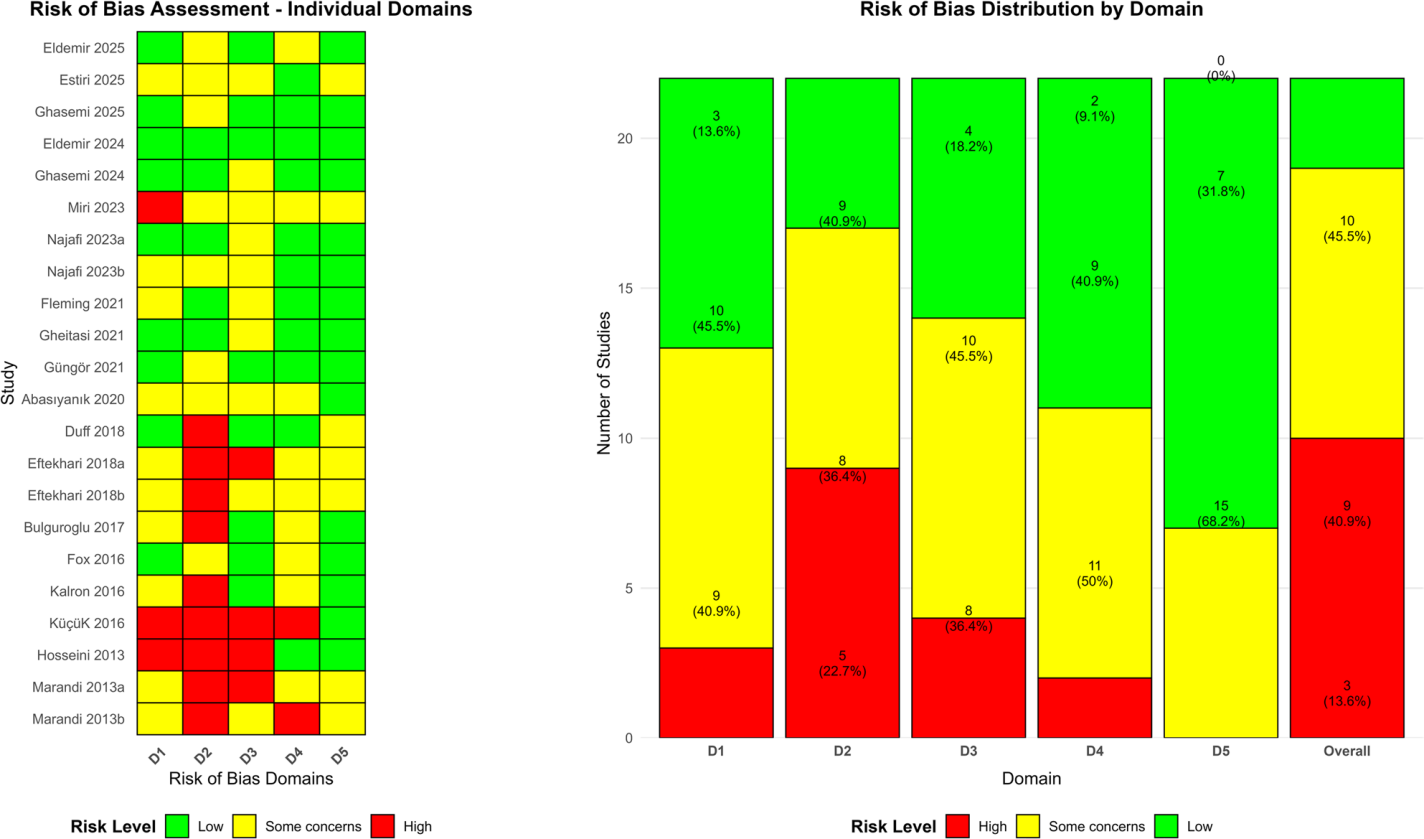
Risk of bias assessment using the RoB 2 tool

Among the 22 RCTs assessed with RoB 2, three (13.6%) were judged low risk of bias overall, nine (40.9%) raised some concerns, and ten (45.5%) were rated high risk. Methodological weaknesses concentrated in three domains: deviations from intended interventions, the randomization process, and missing outcome data. Outcome measurement was generally low risk or some concerns across most studies; reported result selection was predominantly low risk. These findings informed interpretation of the network estimates, SUCRA rankings, and certainty of evidence, and warrant cautious conclusions from the review, as illustrated in Fig. 2.

### Network geometry

Network geometry was constructed to visualize the structure of direct and indirect evidence across included randomized controlled trials for each outcome. Networks spanned multiple Pilates-based modalities and comparator conditions: Pilates, Clinical Pilates, Mat Pilates, Reformer Pilates, Pilates-TR, Pilates Training, Online or Tele-Pilates, Home-Based Pilates, Rebound, Supervised PBCST, Relaxation, Active Control, and standard Control. The standard Control group formed the central hub in most networks, reflecting the predominance of direct comparisons between Pilates-based interventions and usual care, wait-list, or other control conditions.

Each node represents an intervention or comparator, with node size proportional to the number of participants randomized to that node. Edges represent direct head-to-head comparisons, and edge thickness reflects the volume of direct evidence per comparison. Network structures were generally sparse and control-centered, meaning most relative treatment effects derived from comparisons against a common control rather than from extensive direct comparisons between active Pilates modalities.

Given that several networks were sparse and only a limited number contained closed loops, SUCRA rankings were interpreted cautiously. Where no closed loops existed, rankings primarily reflect available direct comparisons against control and should be treated as exploratory rather than definitive evidence of clinical superiority.

Detailed definitions and grouping rationale for each intervention and comparator node are provided in Supplementary Table S3.

#### Network plots

Network plots for each outcome are presented in Figures S1 to S9. The Timed Up and Go network showed one of the more complex structures, with Control as the dominant central node and direct comparisons extending to Pilates, Clinical Pilates, Online Pilates, Mat Pilates, Reformer Pilates, Pilates Training, Rebound, and Supervised PBCST (Figure S1). This network also contained closed loops involving Mat Pilates and Reformer Pilates, and Pilates and Rebound, permitting limited consistency assessment between direct and indirect evidence.

The Berg Balance Scale network was similarly control-centered but included a closed triangular loop between Control, Pilates, and Rebound (Figure S2), with additional direct comparisons between Control and Clinical Pilates, Pilates-TR, and Pilates Training. The 6-Minute Walk Test and 2-Minute Walk Test networks were sparser and predominantly star-shaped. The 6-Minute Walk Test network connected Control directly to Pilates, Pilates-TR, and Pilates Training (Figure S3); the 2-Minute Walk Test network connected Control to Pilates and to Supervised PBCST (Figure S4). Both structures offered limited scope for indirect comparisons beyond the shared control node.

The 10-Meter Walk Test network remained largely control-centered but incorporated a closed loop involving Control, Mat Pilates, and Reformer Pilates, alongside direct comparisons between Control and Pilates, Pilates-TR, and Supervised intervention nodes (Figure S5).

For fatigue outcomes, the Fatigue Severity Scale network took a simple star-shaped form, with Control compared directly against Clinical Pilates, Home-Based Pilates, Pilates, and Pilates Training (Figure S6). The Modified Fatigue Impact Scale network was broader, with Control as the main hub and direct comparisons involving Mat Pilates, Reformer Pilates, Pilates, Pilates-TR, Pilates Training, Tele-Pilates, and Active Control (Figure S7). Closed loops appeared between Control, Mat Pilates, and Reformer Pilates, and between Control, Tele-Pilates, and Active Control.

The MSQOL-54 network was also control-centered, with active nodes including Pilates, Pilates-TR, Mat Pilates, Reformer Pilates, Tele-Pilates, and Active Control (Figure S8). Closed loops mirrored those in the Modified Fatigue Impact Scale network — Mat Pilates and Reformer Pilates, and Tele-Pilates and Active Control — though direct head-to-head evidence across these loops remained limited.

Across all outcomes, the evidence base was dominated by comparisons against control conditions, with only selected outcomes supplying closed loops for indirect comparison. Comparative efficacy estimates across Pilates modalities should therefore be interpreted cautiously, particularly where networks were sparse or star-shaped.

### Network meta-analysis

#### Primary outcomes

##### Timed Up and Go (TUG) test

**Fig. 3:**
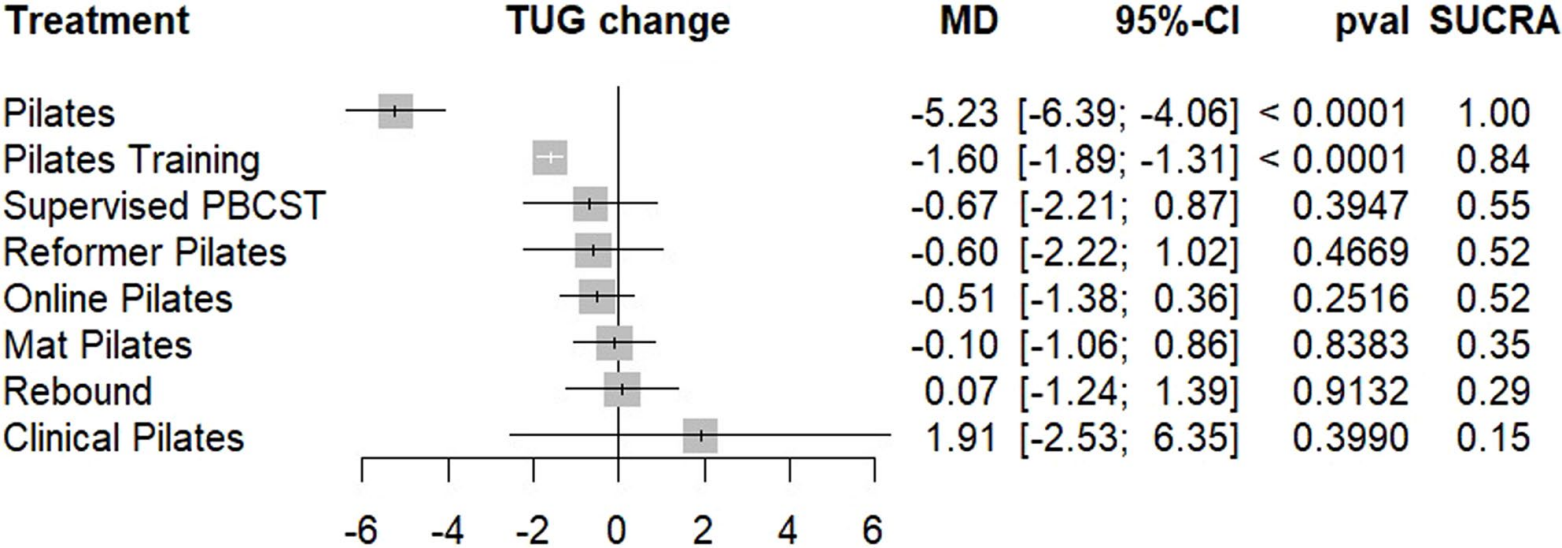
Forest plot of Timed Up and Go (TUG) test change (seconds) for different exercise and control interventions

For the TUG test, where a negative mean difference reflects faster completion and better functional mobility, Pilates produced the largest and statistically significant reduction in completion time versus control (MD = − 5.23 s; 95% CI: −6.39 to − 4.06; *p* < 0.0001) and ranked first across all interventions (SUCRA = 1.00) (Fig. 3). Pilates Training followed with a significant reduction (MD = − 1.60 s; 95% CI: −1.89 to − 1.31; *p* < 0.0001; SUCRA = 0.84).

No other intervention reached statistical significance versus control: Supervised PBCST (MD = − 0.67; 95% CI: −2.21 to 0.87; *p* = 0.3947; SUCRA = 0.55), Reformer Pilates (MD = − 0.60; 95% CI: −2.22 to 1.02; *p* = 0.4669; SUCRA = 0.52), Online Pilates (MD = − 0.51; 95% CI: −1.38 to 0.36; *p* = 0.2516; SUCRA = 0.52), Mat Pilates (MD = − 0.10; 95% CI: −1.06 to 0.86; *p* = 0.8383; SUCRA = 0.35), Rebound (MD = 0.07; 95% CI: −1.24 to 1.39; *p* = 0.9132; SUCRA = 0.29), and Clinical Pilates (MD = 1.91; 95% CI: −2.53 to 6.35; *p* = 0.3990; SUCRA = 0.15). Pilates and Pilates Training were the only interventions associated with meaningful TUG improvement.

#### Secondary outcomes

##### Berg Balance Scale (BBS)

**Fig. 4:**
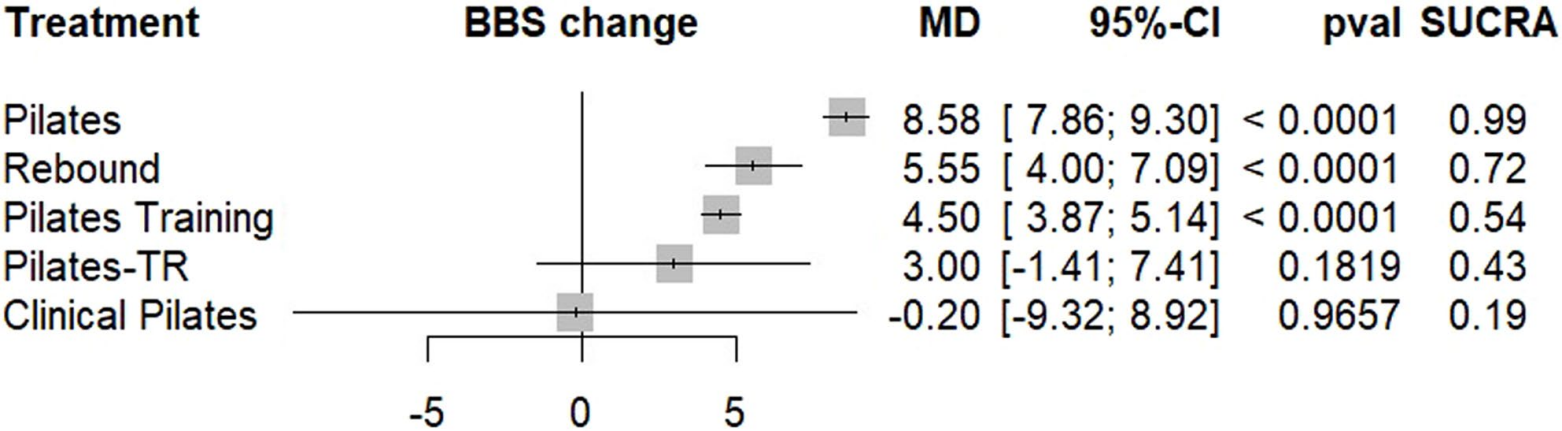
Forest plot of network meta-analysis comparing the effects of different treatments on Berg Balance Scale (BBS) change scores

For the BBS, where a positive mean difference reflects greater balance improvement, Pilates again ranked first and produced the largest significant gain versus control (MD = 8.58 points; 95% CI: 7.86 to 9.30; *p* < 0.0001; SUCRA = 0.99) (Fig. 4). Rebound ranked second with a significant improvement (MD = 5.55 points; 95% CI: 4.00 to 7.09; *p* < 0.0001; SUCRA = 0.72), and Pilates Training ranked third with a comparable significant gain (MD = 4.50 points; 95% CI: 3.87 to 5.14; *p* < 0.0001; SUCRA = 0.54).

Pilates-TR showed a positive but non-significant estimate (MD = 3.00 points; 95% CI: −1.41 to 7.41; *p* = 0.1819; SUCRA = 0.43). Clinical Pilates showed no evidence of benefit (MD = − 0.20 points; 95% CI: −9.32 to 8.92; *p* = 0.9657; SUCRA = 0.19). Pilates, Rebound, and Pilates Training were the three interventions associated with statistically significant BBS improvements, with Pilates carrying the highest ranking probability.

##### 6-Minute Walk Test (6MWT)

**Fig. 5:**
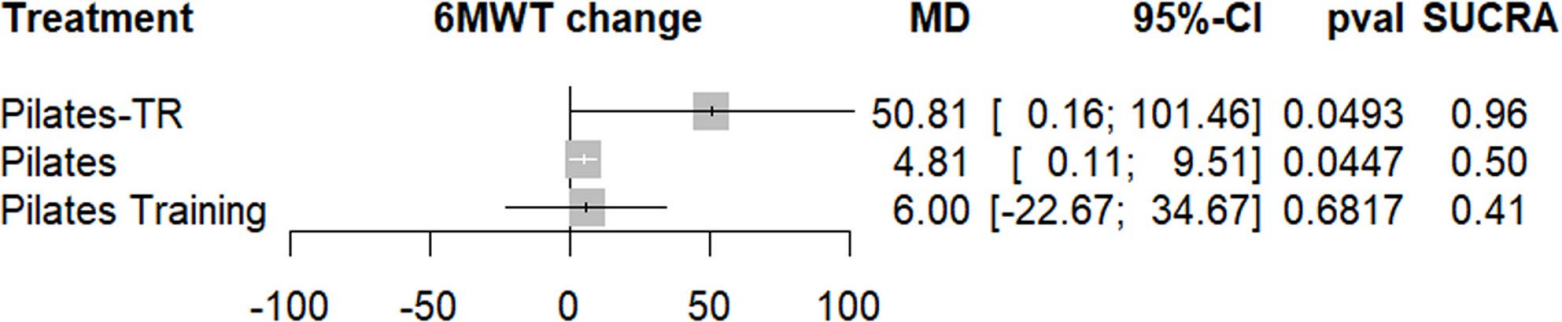
Forest plot comparing the effects of different Pilates-based interventions on 6-Minute Walk Test (6MWT) change

Network meta-analysis of the 6-Minute Walk Test showed that Pilates-TR ranked first (SUCRA = 0.96) with the largest improvement in walking distance versus control (MD = 50.81 m; 95% CI: 0.16 to 101.46; *p* = 0.0493), though the wide confidence interval and borderline *p*-value warrant cautious interpretation (Fig. 5). Pilates also reached significance with a smaller effect (MD = 4.81 m; 95% CI: 0.11 to 9.51; *p* = 0.0447; SUCRA = 0.50). Pilates Training showed no significant difference versus control (MD = 6.00 m; 95% CI: −22.67 to 34.67; *p* = 0.6817; SUCRA = 0.41) (Fig. 5).

##### Modified Fatigue Impact Scale (MFIS)

**Fig. 6:**
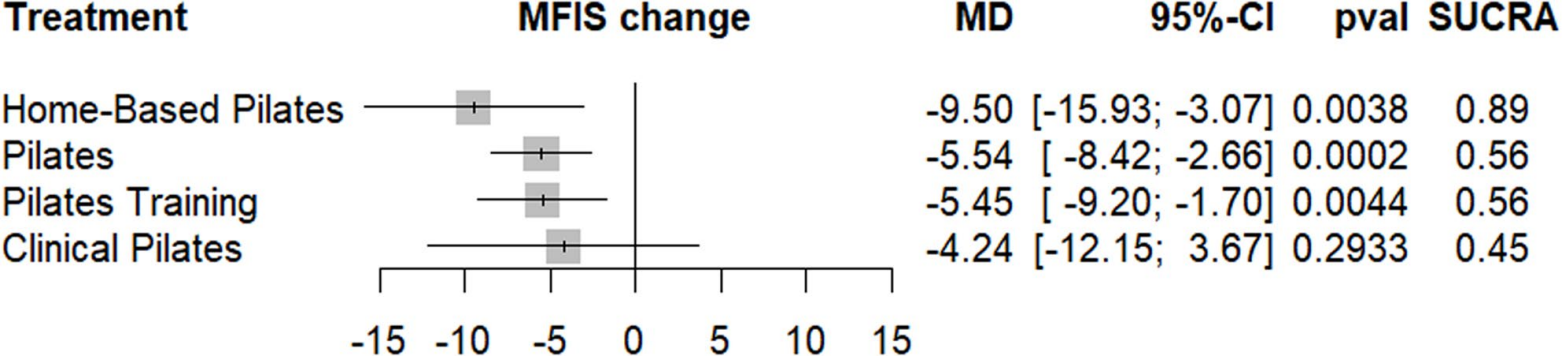
Forest plot comparing the effects of different Pilates-based interventions on Modified Fatigue Impact Scale (MFIS) change

Network meta-analysis of the Modified Fatigue Impact Scale showed that Home-Based Pilates produced the largest statistically significant reduction in fatigue burden versus control (MD = − 9.50 points; 95% CI: −15.93 to − 3.07; *p* = 0.0038; SUCRA = 0.89), followed by Pilates (MD = − 5.54 points; 95% CI: −8.42 to − 2.66; *p* = 0.0002; SUCRA = 0.56) and Pilates Training (MD = − 5.45 points; 95% CI: −9.20 to − 1.70; *p* = 0.0044; SUCRA = 0.56). Clinical Pilates did not reach significance (MD = − 4.24 points; 95% CI: −12.15 to 3.67; *p* = 0.2933; SUCRA = 0.45) (Fig. 6).

##### Fatigue Severity Scale (FSS)

**Fig. 7:**
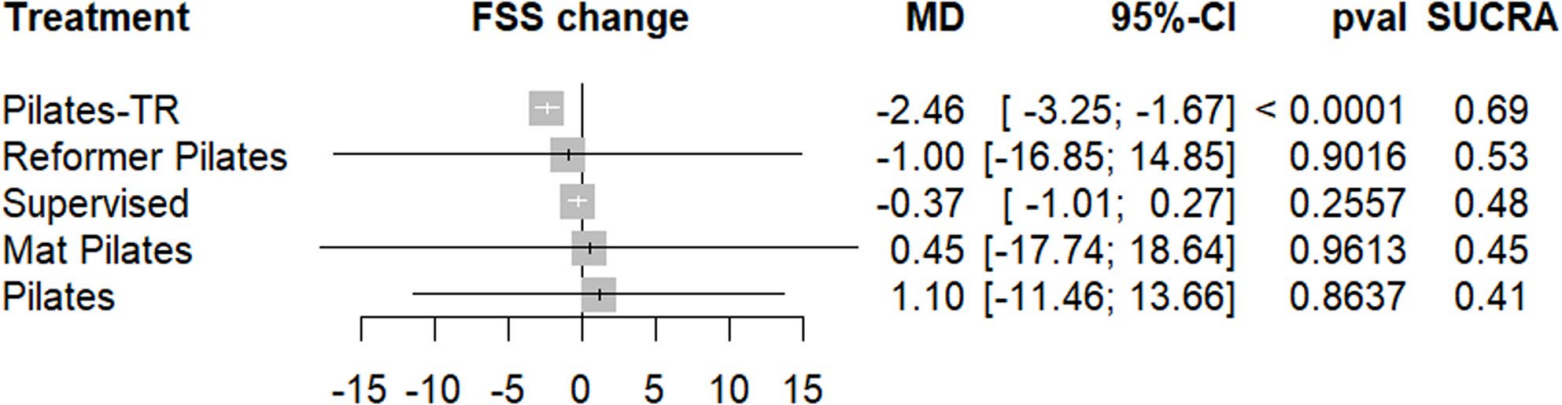
Forest plot comparing the effects of different Pilates-based interventions on Fatigue Severity Scale (FSS) change

Network meta-analysis of the Fatigue Severity Scale showed that Pilates-TR was the only intervention reaching statistical significance versus control (MD = − 2.46 points; 95% CI: −3.25 to − 1.67; *p* < 0.0001; SUCRA = 0.69). All remaining interventions failed to reach significance: Reformer Pilates (MD = − 1.00; 95% CI: −16.85 to 14.85; *p* = 0.9016), Supervised Pilates (MD = − 0.37; 95% CI: −1.01 to 0.27; *p* = 0.2557), with Mat Pilates and Pilates showing nonsignificant positive mean differences of 0.45 and 1.10 points respectively, suggesting no fatigue reduction (Fig. 7).

##### MSQOL-54 physical health

The network meta-analysis assessing the MSQOL-54 Physical Health domain suggested a statistically significant improvement in favor of Pilates-TR compared with control (MD = 17.64 points; 95% CI: 5.97 to 29.31; *p* = 0.0031), as shown in the forest plot (Figure S10). Pilates-TR also had the highest SUCRA value (0.95), followed by Tele-Pilates (MD = 9.40; *p* = 0.0028; SUCRA = 0.72) and Active Control (MD = 7.70; SUCRA = 0.63). However, these rankings should be interpreted cautiously because the network was sparse, and ranking probabilities do not establish definitive clinical superiority without considering effect sizes, confidence intervals, risk of bias, and certainty of evidence.

##### MSQOL-54 mental health

For the MSQOL-54 Mental Health Composite, Pilates-TR was associated with a statistically significant improvement compared with control (MD = 16.55 points; 95% CI: 2.24 to 30.86; *p* = 0.0234), as shown in the forest plot (Figure S11). However, Active Control had the highest SUCRA value (0.84), followed by Pilates-TR (SUCRA = 0.81) and Tele-Pilates (MD = 13.98; *p* = 0.0004; SUCRA = 0.77). Therefore, although Pilates-TR showed a statistically significant effect estimate, it should not be presented as the clearly superior intervention for mental health outcomes. The discordance between the effect estimate and ranking probability reinforces the need to interpret SUCRA rankings alongside confidence intervals, network sparsity, risk of bias, and certainty of evidence.

#### Exploratory walking-performance outcomes

##### 2-Minute walk test

Network meta-analysis of the 2-Minute Walk Test found no statistically significant improvements versus control for any intervention. Supervised PBCST ranked first (SUCRA = 0.82) but did not reach significance (MD = 11.78 m; 95% CI: −7.01 to 30.57; *p* = 0.2191), nor did Pilates (MD = 2.30 m; 95% CI: −21.98 to 26.58; *p* = 0.8527; SUCRA = 0.44). Wide confidence intervals crossing the null limit confidence in both rankings (Fig. S12).

##### 10-Meter walk test

Network meta-analysis of the 10-Meter Walk Test found no Pilates-based intervention reached statistical significance for walking speed. Online Pilates ranked first (SUCRA = 0.96) with the most favorable effect estimate but fell short of significance (MD = 0.18; 95% CI: −0.01 to 0.37; *p* = 0.0618). Pilates showed a small nonsignificant effect (MD = 0.02; 95% CI: −0.09 to 0.13; *p* = 0.6894; SUCRA = 0.58). Relaxation was the only intervention reaching significance, with a statistically significant negative effect (MD = − 0.15; 95% CI: −0.29 to − 0.01; *p* = 0.0356; SUCRA = 0.01) (Fig. S13).

### League tables of pairwise comparisons

League tables summarizing all pairwise treatment comparisons are provided in Table S2, covering TUG, BBS, 6MWT, 2MWT, 10MWT, FSS, MFIS, MSQOL-54 physical health, and MSQOL-54 mental health. Effect estimates are presented as mean differences with 95% confidence intervals, enabling direct comparison between each intervention pair within the network.

The tables complement forest plots and SUCRA rankings by presenting comparative effects between active intervention nodes, not only versus control. For sparse networks with limited direct head-to-head comparisons, estimates are largely indirect and should be interpreted accordingly. The league tables therefore support the overall interpretation of comparative efficacy rather than establish definitive superiority between Pilates modalities.

### Certainty of Evidence (GRADE-NMA)

Supplementary Table 4 summarizes certainty ratings across nine outcomes. No comparison reached high certainty; two were moderate, five low, and two very low. Rankings should be treated as exploratory rather than definitive evidence of superiority.

Pilates ranked first on both functional outcomes (Berg Balance Scale, Timed Up and Go), though low certainty driven by risk of bias and heterogeneity limits confidence in the magnitude of benefit. For walking outcomes, all top-ranked interventions (Pilates-TR, Supervised PBCST, Online Pilates) carried low or very low certainty due to sparse networks, imprecision, and wide confidence intervals; none support reliable claims of comparative superiority.

For fatigue, Pilates-TR showed the most favorable effect on the Fatigue Severity Scale at moderate certainty, while Home-Based Pilates ranked first on the Modified Fatigue Impact Scale at low certainty. For quality of life, Pilates-TR ranked first on the MSQOL-54 Physical Health domain at moderate certainty; Active Control ranked first on the Mental Health domain at low certainty, not any Pilates modality.

Overall, GRADE-NMA supports cautious interpretation of these findings and underscores the need for adequately powered trials with standardized protocols, longer follow-up, and consistent outcome reporting.

### Feasibility of inconsistency, heterogeneity, subgroup, and sensitivity analyses

Formal inconsistency testing was constrained by the sparse, predominantly control-centered network structures. Closed loops were present only in selected outcomes; several networks were star-shaped and relied mainly on direct comparisons against a common control, making local and global inconsistency analyses infeasible for those outcomes. Prespecified subgroup and sensitivity analyses excluding high-risk-of-bias studies were similarly not viable for most outcomes, as too few studies remained within each treatment comparison after stratification or exclusion. These limitations were incorporated into the interpretation of SUCRA rankings and the GRADE-NMA certainty assessments.

## Discussion

Multiple sclerosis (MS) is a chronic, progressive neurodegenerative disease in which long-term exercise programs are essential to maintain or improve functional status [40]. Current evidence supports physical exercise as a key non-pharmacological behavioral intervention with beneficial effects on disease-related symptoms, contributing to the management of functional consequences in MS [41]. As exercise modalities become increasingly popular among this population, rigorous evaluation of their therapeutic benefits is warranted.

Clinical Pilates programs designed and supervised by certified physiotherapists have demonstrated improvements in functional outcomes while enhancing adherence. In parallel, home-based exercise programs offer a cost-effective alternative that is not restricted to a limited intervention period, thereby increasing feasibility and accessibility [32]. Pilates is a low-cost, safe, and appealing exercise modality that integrates flexibility, strength, endurance, mobility, and balance [12, 42]. Clinical Pilates emphasizes core stabilization, posture, and controlled movement through targeted activation of the diaphragm, transversus abdominis, multifidus, and pelvic floor muscles. Consequently, Pilates has been investigated as an alternative or adjunct to Pilates-based telerehabilitation approaches in various chronic conditions to enhance participation and adherence [43, 44].

Cognitive dysfunction represents another major contributor to functional limitations in daily and occupational activities among individuals with MS [45]. Beyond cognitive rehabilitation, exercise training has shown promise in improving cognitive function [46, 47]. Pilates may influence cognitive outcomes because it requires sustained attention to posture, movement control, and breathing. Sosnoff et al. further suggested that home-based exercise programs may be more cost-effective than supervised interventions [48].

In the present study, improvements in mobility, walking speed, and walking endurance following an eight-week Pilates program are consistent with previous findings in people with MS (pwMS) [12, 15, 48, 49]. Accordingly, the purpose of this review was to assess the efficacy of Pilates as a therapeutic intervention for individuals with MS.

The present analysis demonstrated significant improvements in functional mobility following Pilates interventions, particularly as assessed by the Timed Up and Go (TUG) test. These improvements may be attributed to the structured nature of Pilates programs, especially home-based and supervised protocols, which target flexibility, muscle strength, postural stability, and balance. These components are essential for transitional movements and walking performance in people with MS (pwMS). Consistent evidence supports the positive effects of Pilates on functional mobility. Similarly, Abasıyanık et al. demonstrated significant improvements in TUG performance under standard, cognitive, and manual dual-task conditions, indicating that Pilates improves mobility even in cognitively demanding situations [32]. These findings align with the meta-analysis by Sánchez-Lastra et al. which concluded that Pilates significantly improves physical function and balance in pwMS [12], consistent with the results reported by Shahrokhian et al. and Medina-Pérez et al. [50, 51].

Gheitasi et al. observed a reduction in TUG time from 11.7 ± 0.5 s to 9.8 ± 0.4 s following Pilates training, with no significant changes in the control group [30]. Estiri et al. also reported significant improvements in TUG performance, with greater benefits when Pilates was combined with music, suggesting that sensory or motivational factors may enhance training effectiveness [23]. Kalron et al. further confirmed significant improvements in TUG performance in the Pilates group (*p* = 0.023) [15], while Sisi et al. demonstrated that Pilates produced greater mobility improvements than rebound therapy [52].

Beyond mobility outcomes, Pilates has been associated with broader functional improvements. Asvar and Taghian reported significant reductions in physical disability scores following Pilates training [53]. Bulguroğlu et al. demonstrated improvements in functional mobility in both Mat and Reformer Pilates groups, with slightly greater benefits observed in the Reformer-based program [13]. Duff et al. also reported significant improvements in walking ability following Pilates training, underscoring its relevance to daily functional activities [33].

Although no studies have directly evaluated the effects of Pilates on core power and endurance in MS or other neurological populations, increased core stability, particularly through activation of the transversus abdominis, may explain these functional gains. Centering, a fundamental principle of Pilates, activates the transversus abdominis, leading to tensioning of the thoracolumbar fascia and enhanced trunk stability.

Supervision appears to play a critical role in optimizing outcomes. Eldemir et al. reported significant improvements in muscle strength across the shoulder, hip, knee, and ankle joints, as well as enhanced core endurance measured by side-bridge and trunk flexor tests, following supervised Pilates combined with Pilates-based telerehabilitation delivered via videoconferencing [14]. These improvements were accompanied by subsequent enhancements in TUG performance [22]. Improvements in walking ability may therefore be mediated by increased muscle strength, improved balance, and reduced fatigue.

Additional evidence supports these findings. Küçük et al. reported improvements in TUG turning components [37], while Marandi et al. demonstrated that both Pilates and aquatic training significantly improved mobility outcomes [39].

In contrast, Fox et al. and Güngör et al. did not observe significant differences between Pilates and other exercise modalities [35, 54], potentially due to variations in intervention intensity, supervision, or participant characteristics. The available evidence suggests Pilates may improve functional mobility in people with MS, though confidence in this finding remains limited by risk of bias, sparse network structures, and heterogeneity across intervention protocols. From a theoretical perspective, dynamic systems theory posits that postural control arises from the interaction, synchronization, and integration of the nervous, muscular, and skeletal systems, collectively referred to as the postural control system [49]. This system requires effective balance and movement control to integrate sensory information and determine body position in space, as well as sufficient musculoskeletal capacity to generate appropriate force. Musculoskeletal factors influencing balance regulation include muscle strength, endurance, range of motion, and biomechanical alignment, while the nervous system plays a central role in coordinating postural responses. Accordingly, exercise approaches such as Pilates, which promote coordination, integration, and convergence of these systems, may lead to improvements in balance, mobility, and motor control.

Regarding walking endurance, our analysis demonstrated that Pilates combined with Pilates-based telerehabilitation (Pilates-TR) resulted in significant improvements in the 6-Minute Walk Test (6MWT). This finding is consistent with reports by Abasıyanık et al. [32], Duff et al. [33], Eldemir et al. [22], and Miri et al. [26], all of whom observed significant post-intervention gains in walking endurance following Pilates-based programs. Integrating Pilates with conventional rehabilitation appears more advantageous than practicing Pilates in isolation. Pilates-based telerehabilitation initially targets specific impairments and pathological constraints through comprehensive assessment, pain management, restoration of joint mobility, and individualized treatment planning. In contrast, Pilates primarily enhances functional movement quality, muscular strength, core stability, and proprioception. Conventional therapy therefore identifies and corrects movement dysfunctions and structural limitations that Pilates alone may not fully address. When Pilates-based exercises are introduced after or alongside conventional rehabilitation, patients benefit from both therapeutic correction and improved neuromuscular control. This sequential and complementary approach facilitates greater walking endurance and functional performance, as reflected by improvements in the 6MWT. Compared with isolated Pilates practice, which may lack clinical tailoring, this integrated model better aligns therapeutic goals with progressive strengthening and movement retraining. Moreover, the supervision inherent to Pilates-based telerehabilitation ensures that exercises remain safe, individualized, and biomechanically correct. Such oversight improves movement quality and prevents compensatory patterns that can limit training effectiveness. Pilates subsequently builds on these corrected movement patterns to enhance strength, stability, and endurance in a manner that directly transfers to walking-related tasks such as the 6MWT. Thus, the observed benefit is not merely attributable to increased exercise volume, but rather to the combination of targeted remedial intervention followed by structured, controlled, and functionally relevant movement practice under appropriate clinical supervision.

Güngör et al. observed improvements in both TUG and 2-Minute Walk Test (2MWT) outcomes, with larger effect sizes in supervised Pilates groups [54]. Kalron et al. also demonstrated improvements in Multiple Sclerosis Walking Scale-12 (MSWS-12) and 2MWT performance following Pilates training [15]. Recent evidence supports the effectiveness of remotely delivered Pilates interventions. Najafi et al. reported significant improvements in walking speed following tele-Pilates, with superior T25FW outcomes compared with tele-yoga [27, 28]. However, the available evidence remains limited, and one identified study was available only as a protocol without reported outcomes. Similarly, Rimmer et al. found significant mobility improvements following both tele-Pilates and clinic-based Pilates programs [55]. Improvements in gait performance may be mediated by feedforward activation of core musculature, which enhances trunk stability and reduces center-of-gravity displacement during ambulation [56, 57]. Additionally, Pilates may improve sensorimotor integration through its emphasis on torso control and neutral spine alignment [8, 58].

Pilates ranked highly for balance on the Berg Balance Scale, but this ranking should be interpreted alongside the effect estimates, confidence intervals, risk of bias, network geometry, and GRADE-NMA certainty ratings. Improvements in balance may be attributed to increased core strength, tactile and verbal feedback, enhanced body awareness, and improved neuromuscular coordination [34]. Abasıyanık et al. reported significant improvements in balance confidence measured by the Activities-specific Balance Confidence (ABC) scale [59], while objective measures, including limits of stability and mediolateral control, also improved significantly [32]. Similar findings were reported by Asvar and Taghian [53], Bulguroğlu et al. [13], and Nilsagård et al. [60].

Randomized controlled trials by Eldemir et al. demonstrated significant improvements in BBS scores, walking speed, and postural stability following Pilates interventions [14, 22]. Estiri et al. reported reductions in postural sway assessed by stabilimetric analysis [23], and Gheitasi et al. observed increased BBS scores after Pilates training [30]. Soysal et al. reported improvements in sensory integration during balance testing, indicating enhanced proprioceptive control [54]. Additional balance improvements were reported by Sisi et al. [52], Kalron et al. [15], Küçük et al. [37], Marandi et al. [39], and Rimmer et al. [55].

Mechanistic evidence further supports these findings. Studies by Freeman et al., Cruz-Ferreira et al., Herrington et al., and Critchley et al. highlight the role of deep abdominal muscle activation, particularly the transversus abdominis, in balance and postural control [61–64]. Freeman et al. suggested that improvements in balance and mobility may be mediated by enhanced activation of deep trunk musculature responsible for core stabilization [61]. Cruz-Ferreira et al. reported limited evidence regarding transversus abdominis and obliquus internus activation during Pilates exercises in healthy adults [62], whereas Herrington et al. demonstrated increased transversus abdominis contraction following Pilates training in asymptomatic individuals [63]. Critchley et al. further confirmed changes in transversus abdominis and obliquus internus activation using ultrasonography after an eight-week Pilates program delivered twice weekly [64].

Conflicting findings reported by Duff et al. [33] and Kileff et al. [65] may be explained by differences in disease stage, lesion burden, or exercise dosage. Moreover, brain volume reduction observed in later stages of MS may contribute to impaired postural control and cognitive decline, potentially limiting responsiveness to exercise-based interventions [66].

Balance performance in pwMS is closely associated with trunk and lower-limb muscle strength. Increased strength in these muscle groups has been shown to enhance physical mobility and balance [67]. DeBolt et al. reported inconsistent results, likely due to the specific characteristics, intensity, and focus of their exercise program, which emphasized isolated lower trunk strengthening [68]. In contrast, the studies by Filipi et al. and the present review emphasize comprehensive strengthening approaches that target general muscular strength, resulting in improved motor function [69].

Pilates exercises, which promote progressive strengthening, are therefore considered effective for improving postural stability by enhancing lower-limb muscle strength and core stability [70]. Progressive resistance training may also improve joint position sense by increasing muscle spindle sensitivity, which likely contributed to the significant balance improvements observed compared with control groups. Given the reliance of lower-limb function on proximal stability and the central role of balance impairment in MS, Pilates may effectively strengthen lower-limb musculature, particularly extensor and flexor muscle groups, thereby supporting postural control [70].

Finally, Johnson et al. reported that error correction and tactile cueing during exercise enhance kinesthetic awareness, further contributing to balance improvements [71]. Collectively, these findings suggest that improvements in balance following Pilates training may result from enhanced physical fitness, increased muscular strength and endurance, particularly of the core, and improved sensorimotor integration.

Pilates-based interventions were associated with reductions in fatigue severity and impact across several studies, though certainty and consistency varied by fatigue instrument and intervention format. Improvements were reported by Asvar and Taghian [53], Bulguroğlu et al. [13], Eldemir et al. [14, 22], Fleming et al. [29], Küçük et al. [37], and Salehzadeh et al. [72]. The consistency of these findings may be partly explained by intervention duration and training frequency. Supervised and tele-Pilates programs generally produced larger effects than unsupervised home-based interventions [54, 55]. Although Duff et al. and Kalron et al. reported neutral findings [15, 33], Miri et al. demonstrated significant reductions in fatigue using the Fatigue Impact Scale [26]. Improvements in fatigue may reflect enhanced muscle efficiency and reduced energy expenditure during functional activities [22].

Improvements in health-related quality of life were observed in some Pilates-based intervention groups, particularly for physical health outcomes; however, the mental health ranking results were less consistent and should be interpreted cautiously because Active Control had the highest SUCRA value in that network. Abasıyanık et al. [59], Bulguroğlu et al. [13], Eldemir et al. [14], Estiri et al. [23], Miri et al. [26], and Najafi et al. [27, 28] all reported improvements using validated quality-of-life instruments. The consistency of these findings across different assessment tools strengthens the evidence that Pilates confers multidimensional benefits, extending beyond physical function to encompass psychological and emotional well-being.

In contrast, Baquet et al. and Duff et al. reported nonsignificant findings, which may be attributed to low exercise intensity and small sample sizes [33, 73]. Previous studies suggest that improvements in quality of life among people with MS may result from exercise-induced effects on both motor and neuropsychological systems [74, 75]. In line with the present findings, reductions in physical pain and perceived physical and mental limitations following Pilates training were associated with improvements in overall mental and physical health, thereby contributing to enhanced quality of life.

The pathophysiology of fatigue in MS remains incompletely understood, and further studies are required to clarify the mechanisms underlying fatigue reduction following exercise interventions. Nevertheless, Roshandelpour et al. proposed that increased metabolic activity during and after exercise may partially explain improvements in fatigue levels [75]. One potential explanation for the effectiveness of Pilates in improving mental quality of life is the enhancement of self-confidence and perceived competence in managing daily challenges.

However, the lack of significant improvement in certain physical quality-of-life domains observed in some studies may be attributable to several factors. First, rapid disease progression prior to study enrollment may have limited the responsiveness to an eight-week intervention, suggesting that longer-duration programs may be necessary to achieve measurable physical benefits. Second, the content of Pilates interventions, often emphasizing psychological components such as concentration, breathing, and relaxation while avoiding high-risk movements due to participant vulnerability, may not have provided sufficient physical stimulus to elicit substantial improvements in physical capacity.

This network meta-analysis has several limitations. Considerable heterogeneity existed across studies regarding Pilates protocols, including variations in intensity, duration, frequency, supervision, and delivery mode, which may have influenced effect estimates and limited comparability. Outcome measures for fatigue, balance, and quality of life were not uniform, potentially introducing measurement inconsistency. Many studies had small sample sizes and short intervention periods, reducing statistical power and limiting assessment of long-term effects. Blinding of participants and therapists was generally not feasible, increasing the risk of performance and detection bias. Adherence was inconsistently reported, particularly in home-based and tele-rehabilitation programs. Additionally, most studies included participants with mild to moderate disability, restricting generalizability to those with advanced multiple sclerosis, and long-term follow-up data were scarce.

Future research should include adequately powered randomized trials with standardized Pilates protocols, clear reporting of dosage, supervision, and adherence, and longer follow-up to assess sustainability of effects on fatigue, balance, and quality of life. Studies should also examine optimal integration with Pilates-based telerehabilitation, cost-effectiveness, and comparative efficacy across disability levels. Given the growing evidence for tele-Pilates, implementation, acceptability, adherence, and digital monitoring warrant further investigation. Supervised Pilates may serve as an individualized adjunct to rehabilitation for selected people with multiple sclerosis, particularly when delivered by trained physiotherapists. Routine implementation, however, should await adequately powered trials with standardized protocols, longer follow-up, formal certainty assessment, and clearer evidence of clinically meaningful benefit.

Pilates-based interventions may offer benefits across functional mobility, balance, walking endurance, fatigue, and health-related quality of life in people with MS, but no outcome reached high certainty. Findings were limited by risk of bias, heterogeneity across protocols, sparse network structures, imprecision, and short follow-up. Rankings for Pilates, Pilates combined with Pilates-based telerehabilitation, home-based Pilates, and tele-Pilates should be treated as exploratory rather than evidence of comparative superiority. In clinical practice, supervised Pilates may serve as an individualized adjunct to rehabilitation for selected patients, particularly when delivered by trained physiotherapists and tailored to disability level, fatigue burden, and functional goals. Routine implementation should await adequately powered trials with standardized protocols, consistent outcome measures, and longer follow-up to confirm clinically meaningful and durable benefits.

## Supplementary Information

Supplementary Material 1.

Supplementary Material 2.

Supplementary Material 3.

Supplementary Material 4.

Supplementary Material 5.

Supplementary Material 6.

## Data Availability

All data generated or analysed during this study are included in this published article and its supplementary information files. The R code used for the analyses is available from the corresponding author upon reasonable request.

## Abbreviations

MS: Multiple Sclerosis
TUG: Timed Up and Go
BBS: Berg Balance Scale
6MWT: 6-Minute Walk Test
2MWT: 2-Minute Walk Test
FSS: Fatigue Severity Scale
MFIS: Modified Fatigue Impact Scale
MSQOL-54: Multiple Sclerosis Quality of Life-54
NMA: Network Meta-Analysis
SUCRA: Surface Under the Cumulative Ranking Curve
