# Supplementary material for: Comparative effects of pilates-based interventions on functional mobility, balance, fatigue, and quality of life in people with multiple sclerosis: a systematic review and network meta-analysis
Source: BMC Sports Sci Med Rehabil. 2026 Jul 4;18:307. doi: 10.1186/s13102-026-01827-1 (PMC13340119; doi:10.1186/s13102-026-01827-1)
Supplement: Supplementary file 1 — Supplementary Material 1. [file 13102_2026_1827_MOESM1_ESM.docx]

**Supplementary Table 1:** Detailed search strategy

| **Databases** | Boolean search strategy | No. of results |
| --- | --- | --- |
| **PubMed** | ("Multiple Sclerosis"[Mesh] OR "Multiple Sclerosis" OR "MS" OR "Demyelinating Autoimmune Diseases, CNS"[Mesh] OR "Demyelinating Diseases"[Mesh])  AND  ("Pilates"[Mesh] OR "Pilates" OR "Pilates Training" OR "Pilates Exercise" OR "Pilates Method" OR "Pilates-Based" OR "Mat Pilates" OR "Reformer Pilates" OR "Clinical Pilates" OR "Tele-Pilates" OR "Telerehabilitation"[Mesh] OR "Telemedicine"[Mesh]) | 68 |
| **Cochrane** | (Multiple Sclerosis OR MS OR Demyelinating Diseases)  AND  (Pilates OR "Pilates Training" OR "Pilates Exercise" OR "Pilates Method" OR "Pilates-Based" OR "Mat Pilates" OR "Reformer Pilates" OR "Clinical Pilates" OR "Tele-Pilates" OR Telerehabilitation OR Telemedicine) | 102 |
| **Scopus** | (TITLE-ABS-KEY ("Multiple Sclerosis" OR "MS" OR "demyelinating disease"))  AND  (TITLE-ABS-KEY (Pilates OR "Pilates Training" OR "Pilates Exercise" OR "Pilates Method" OR "Pilates-Based" OR "Mat Pilates" OR "Reformer Pilates" OR "Clinical Pilates" OR "Tele-Pilates" OR telerehabilitation OR telemedicine)) | 89 |
| **Web of Science** | TS = ("Multiple Sclerosis" OR "MS" OR "demyelinating disease")  AND  TS = (Pilates OR "Pilates Training" OR "Pilates Exercise" OR "Pilates Method" OR "Pilates-Based" OR "Mat Pilates" OR "Reformer Pilates" OR "Clinical Pilates" OR "Tele-Pilates" OR telerehabilitation OR telemedicine) | 133 |
