# Supplementary material for: Comparative effects of pilates-based interventions on functional mobility, balance, fatigue, and quality of life in people with multiple sclerosis: a systematic review and network meta-analysis
Source: BMC Sports Sci Med Rehabil. 2026 Jul 4;18:307. doi: 10.1186/s13102-026-01827-1 (PMC13340119; doi:10.1186/s13102-026-01827-1)
Supplement: Supplementary file 2 — Supplementary Material 2. [file 13102_2026_1827_MOESM2_ESM.docx]

**Supplementary Table 2:** League Table of Network Meta-Analysis for Comparative Effectiveness of Pilates-Based Interventions on Various Outcome Measures in Multiple Sclerosis

| **Timed Up and Go (TUG) Test** | | | | | | | | | | | | | | | | |
| --- | --- | --- | --- | --- | --- | --- | --- | --- | --- | --- | --- | --- | --- | --- | --- | --- |
| **Clinical Pilates** | 1.91 ( -2.53; 6.35) | . | . | . | | . | | . | | . | | . | |  |  |  |
| 1.91 ( -2.53; 6.35) | **Control** | 0.10 ( -0.86; 1.06) | 0.51 ( -0.36; 1.38) | 5.23 ( 4.06; 6.39) | | 1.60 ( 1.31; 1.89) | | 1.44 ( 0.08; 2.80) | | 0.60 ( -1.02; 2.22) | | 0.67 ( -0.87; 2.21) | |  |  |  |
| 2.01 ( -2.53; 6.56) | 0.10 ( -0.86; 1.06) | **Mat Pilates** | . | . | | . | | . | | 0.50 ( -1.22; 2.22) | | . | |  |  |  |
| 2.42 ( -2.11; 6.95) | 0.51 ( -0.36; 1.38) | 0.41 ( -0.89; 1.71) | **Online Pilates** | . | | . | | . | | . | | . | |  |  |  |
| 7.14 ( 2.55;11.73) | 5.23 ( 4.06; 6.39) | 5.13 ( 3.62; 6.63) | 4.72 ( 3.26; 6.17) | **Pilates** | | . | | -8.96 (-10.70;-7.22) | | . | | . | |  |  |  |
| 3.51 ( -0.94; 7.96) | 1.60 ( 1.31; 1.89) | 1.50 ( 0.50; 2.50) | 1.09 ( 0.17; 2.01) | -3.63 ( -4.82;-2.43) | | **Pilates Training** | | . | | . | | . | |  |  |  |
| 1.84 ( -2.79; 6.47) | -0.07 ( -1.39; 1.24) | -0.17 ( -1.80; 1.45) | -0.58 ( -2.16; 0.99) | -5.30 ( -6.81;-3.79) | | -1.67 ( -3.02;-0.33) | | **Rebound** | | . | | . | |  |  |  |
| 2.51 ( -2.22; 7.24) | 0.60 ( -1.02; 2.22) | 0.50 ( -1.22; 2.22) | 0.09 ( -1.75; 1.93) | -4.63 ( -6.62;-2.63) | | -1.00 ( -2.64; 0.64) | | 0.67 ( -1.41; 2.76) | | **Reformer Pilates** | | . | |  |  |  |
| 2.58 ( -2.12; 7.28) | 0.67 ( -0.87; 2.21) | 0.57 ( -1.25; 2.39) | 0.16 ( -1.61; 1.93) | -4.56 ( -6.49;-2.62) | | -0.93 ( -2.50; 0.64) | | 0.74 ( -1.28; 2.77) | | 0.07 ( -2.16; 2.30) | | **Supervised PBCST** | |  |  |  |
|  |  |  |  |  |  |  |  |  |  |  |  |  |  |  |  |  |
|  |  |  |  |  | |  | |  | |  | |  | |  | |  |
| **Berg Balance Scale (BBS)** | | | | | | | | | | | | | | | | |
| **Clinical Pilates** | -0.20 (-9.32; 8.92) | . | . | . | | . | |  | |  | |  | |  | |  |
| -0.20 ( -9.32; 8.92) | **Control** | -8.58 (-9.30;-7.86) | -3.00 (-7.41; 1.41) | -4.50 (-5.14;-3.87) | | -3.44 (-5.16;-1.72) | |  | |  | |  | |  | |  |
| -8.78 (-17.92; 0.37) | -8.58 ( -9.30;-7.86) | **Pilates** | . | . | | -0.01 (-1.92; 1.90) | |  | |  | |  | |  | |  |
| -3.20 (-13.32; 6.92) | -3.00 ( -7.41; 1.41) | 5.58 ( 1.11;10.04) | **Pilates-TR** | . | | . | |  | |  | |  | |  | |  |
| -4.70 (-13.84; 4.43) | -4.50 ( -5.14;-3.87) | 4.07 ( 3.11; 5.04) | -1.50 ( -5.95; 2.95) | **Pilates Training** | | . | |  | |  | |  | |  | |  |
| -5.75 (-14.99; 3.50) | -5.55 ( -7.09;-4.00) | 3.03 ( 1.45; 4.60) | -2.55 ( -7.22; 2.12) | -1.05 ( -2.72; 0.62) | | **Rebound** | |  | |  | |  | |  | |  |
|  |  |  |  |  | |  | |  | |  | |  | |  | |  |
| **6-Minute Walk Test (6MWT)** | | | | | | | | | | | | | | | | |
| **Control** | -4.81 ( -9.51; -0.11) | -50.81 (-101.46; -0.16) | -6.00 ( -34.67; 22.67) |  |  | |  | |  | |  | |  |  |  |  |
| -4.81 ( -9.51; -0.11) | **Pilates** | . | . |  |  | |  | |  | |  | |  |  |  |  |
| -50.81 (-101.46; -0.16) | -46.00 ( -96.86; 4.87) | **Pilates-TR** | . |  |  | |  | |  | |  | |  |  |  |  |
| -6.00 ( -34.67; 22.67) | -1.19 ( -30.24; 27.87) | 44.81 ( -13.39;103.01) | **Pilates Training** |  |  | |  | |  | |  | |  |  |  |  |
|  |  |  |  |  |  | |  | |  | |  | |  |  |  |  |
|  |  |  |  |  | |  | |  | |  | |  | |  | |  |
| **2-Minute Walk Test (2MWT)** | | | | | | | | | | | | | | | | |
| **Control** | -2.30 (-26.58;21.98) | -11.78 (-30.57; 7.01) |  |  | |  | |  | |  | |  | |  | |  |
| -2.30 (-26.58;21.98) | **Pilates** | . |  |  | |  | |  | |  | |  | |  | |  |
| -11.78 (-30.57; 7.01) | -9.48 (-40.18;21.22) | **Supervised PBCST** |  |  | |  | |  | |  | |  | |  | |  |
|  |  |  |  |  | |  | |  | |  | |  | |  | |  |
| **10 Meter Walk Test (10MWT)** | | | | | | | | | | | | | | | | |
| **Control** | -0.18 (-0.37; 0.01) | -0.02 (-0.13; 0.09) | 0.27 ( 0.12; 0.42) |  | |  | |  | |  | |  | |  | |  |
| -0.18 (-0.37; 0.01) | **Online Pilates** | . | . |  | |  | |  | |  | |  | |  | |  |
| -0.02 (-0.13; 0.09) | 0.16 (-0.06; 0.38) | **Pilates** | 0.06 (-0.09; 0.21) |  | |  | |  | |  | |  | |  | |  |
| 0.15 ( 0.01; 0.29) | 0.33 ( 0.10; 0.57) | 0.17 ( 0.03; 0.32) | **Relaxation** |  | |  | |  | |  | |  | |  | |  |
|  |  |  |  |  | |  | |  | |  | |  | |  | |  |
|  |  |  |  |  | |  | |  | |  | |  | |  | |  |
|  |  |  |  |  | |  | |  | |  | |  | |  | |  |
| **Fatigue Severity Scale (FSS)** | | | | | | | | | | | | | | | | |
| **Control** | -0.45 (-18.64;17.74) | -1.10 (-13.66;11.46) | 2.46 ( 1.67; 3.25) | 1.00 (-14.85;16.85) | | 0.37 ( -0.27; 1.01) | |  | |  | |  | |  | |  |
| -0.45 (-18.64;17.74) | **Mat Pilates** | . | . | 1.45 (-10.85;13.75) | | . | |  | |  | |  | |  | |  |
| -1.10 (-13.66;11.46) | -0.65 (-22.76;21.46) | **Pilates** | . | . | | . | |  | |  | |  | |  | |  |
| 2.46 ( 1.67; 3.25) | 2.91 (-15.30;21.12) | 3.56 ( -9.03;16.15) | **Pilates-TR** | . | | . | |  | |  | |  | |  | |  |
| 1.00 (-14.85;16.85) | 1.45 (-10.85;13.75) | 2.10 (-18.12;22.32) | -1.46 (-17.33;14.41) | **Reformer Pilates** | | . | |  | |  | |  | |  | |  |
| 0.37 ( -0.27; 1.01) | 0.82 (-17.38;19.02) | 1.47 (-11.11;14.05) | -2.09 ( -3.11;-1.07) | -0.63 (-16.49;15.23) | | **Supervised PBCST** | |  | |  | |  | |  | |  |
|  |  |  |  |  | |  | |  | |  | |  | |  | |  |
| **Modified Fatigue Impact Scale (MFIS)** | | | | | | | | | | | | | | | | |
| **Clinical Pilates** | -4.24 (-12.15; 3.67) | . | . | . | |  | |  | |  | |  | |  | |  |
| -4.24 (-12.15; 3.67) | **Control** | 9.50 ( 3.07;15.93) | 5.54 ( 2.66; 8.42) | 5.45 ( 1.70; 9.20) | |  | |  | |  | |  | |  | |  |
| 5.26 ( -4.93;15.45) | 9.50 ( 3.07;15.93) | **Home-Based Pilates** | . | . | |  | |  | |  | |  | |  | |  |
| 1.30 ( -7.12; 9.72) | 5.54 ( 2.66; 8.42) | -3.96 (-11.01; 3.09) | **Pilates** | . | |  | |  | |  | |  | |  | |  |
| 1.21 ( -7.54; 9.96) | 5.45 ( 1.70; 9.20) | -4.05 (-11.49; 3.39) | -0.09 ( -4.82; 4.64) | **Pilates Training** | |  | |  | |  | |  | |  | |  |
|  |  |  |  |  | |  | |  | |  | |  | |  | |  |
| **MSQOL-54 Physical Health** | | | | | | | | | | | | | | | | |
| **Active Control** | 7.70 ( 1.82;13.58) | . | . | . | | . | | . | | -1.70 ( -7.49; 4.09) | |  | |  | |  |
| 7.70 ( 1.82;13.58) | **Control** | -1.00 (-14.03;12.03) | -2.20 (-15.69;11.29) | -17.64 (-29.31;-5.97) | | -5.60 (-10.54;-0.66) | | -3.82 (-14.69; 7.05) | | -9.40 (-15.56;-3.24) | |  | |  | |  |
| 6.70 ( -7.60;21.00) | -1.00 (-14.03;12.03) | **Mat Pilates** | . | . | | . | | -2.82 (-11.72; 6.08) | | . | |  | |  | |  |
| 5.50 ( -9.22;20.22) | -2.20 (-15.69;11.29) | -1.20 (-19.96;17.56) | **Pilates** | . | | . | | . | | . | |  | |  | |  |
| -9.94 (-23.01; 3.13) | -17.64 (-29.31;-5.97) | -16.64 (-34.14; 0.86) | -15.44 (-33.28; 2.40) | **Pilates-TR** | | . | | . | | . | |  | |  | |  |
| 2.10 ( -5.58; 9.78) | -5.60 (-10.54;-0.66) | -4.60 (-18.54; 9.34) | -3.40 (-17.77;10.97) | 12.04 ( -0.63;24.71) | | **Pilates Training** | | . | | . | |  | |  | |  |
| 3.88 ( -8.48;16.24) | -3.82 (-14.69; 7.05) | -2.82 (-11.72; 6.08) | -1.62 (-18.94;15.70) | 13.82 ( -2.13;29.77) | | 1.78 (-10.16;13.72) | | **Reformer Pilates** | | . | |  | |  | |  |
| -1.70 ( -7.49; 4.09) | -9.40 (-15.56;-3.24) | -8.40 (-22.81; 6.01) | -7.20 (-22.03; 7.63) | 8.24 ( -4.95;21.43) | | -3.80 (-11.69; 4.09) | | -5.58 (-18.07; 6.91) | | **Tele-Pilates** | |  | |  | |  |
|  |  |  |  |  | |  | |  | |  | |  | |  | |  |
|  |  |  |  |  | |  | |  | |  | |  | |  | |  |
| **MSQOL-54 Mental Health** | | | | | | | | | | | | | | | | |
| **Active Control** | 15.86 ( 9.20;22.52) | . | . | . | | . | | 1.88 ( -3.97; 7.73) | |  | |  | |  | |  |
| 15.86 ( 9.20;22.52) | **Control** | -2.24 (-14.85;10.37) | -1.70 (-13.86;10.46) | -16.55 (-30.86;-2.24) | | -5.60 (-16.33; 5.13) | | -13.98 (-21.73;-6.23) | |  | |  | |  | |  |
| 13.62 ( -0.64;27.88) | -2.24 (-14.85;10.37) | **Mat Pilates** | . | . | | -3.36 (-12.09; 5.37) | | . | |  | |  | |  | |  |
| 14.16 ( 0.30;28.02) | -1.70 (-13.86;10.46) | 0.54 (-16.98;18.06) | **Pilates** | . | | . | | . | |  | |  | |  | |  |
| -0.69 (-16.47;15.09) | -16.55 (-30.86;-2.24) | -14.31 (-33.38; 4.76) | -14.85 (-33.62; 3.92) | **Pilates-TR** | | . | | . | |  | |  | |  | |  |
| 10.26 ( -2.37;22.89) | -5.60 (-16.33; 5.13) | -3.36 (-12.09; 5.37) | -3.90 (-20.12;12.32) | 10.95 ( -6.93;28.83) | | **Reformer Pilates** | | . | |  | |  | |  | |  |
| 1.88 ( -3.97; 7.73) | -13.98 (-21.73;-6.23) | -11.74 (-26.54; 3.06) | -12.28 (-26.70; 2.14) | 2.57 (-13.70;18.84) | | -8.38 (-21.62; 4.86) | | **Tele-Pilates** | |  | |  | |  | |  |
