# Supplementary material for: Comparative effects of pilates-based interventions on functional mobility, balance, fatigue, and quality of life in people with multiple sclerosis: a systematic review and network meta-analysis
Source: BMC Sports Sci Med Rehabil. 2026 Jul 4;18:307. doi: 10.1186/s13102-026-01827-1 (PMC13340119; doi:10.1186/s13102-026-01827-1)
Supplement: Supplementary file 3 — Supplementary Material 3. [file 13102_2026_1827_MOESM3_ESM.docx]

**Supplementary Table S4:** Node definitions used in the network meta-analysis

| **Node** | **Definition** | **Included arms** | **Rationale** |
| --- | --- | --- | --- |
| Control | Inactive, usual-care, wait-list, or comparator condition without a Pilates-specific intervention. | Control; Control Group; Control (Pre/Post); Wait-List Control; Home Exercise when used as the comparator arm. | Used as the common reference node to connect Pilates modalities across sparse outcome networks. Comparator intensity should be acknowledged when interpreting effects. |
| Active Control | Structured non-Pilates active comparator involving exercise, rehabilitation, or mind-body training. | Tele-Yoga; Yoga; Physical Therapy; Standardized Exercises when coded as an active comparator. | Kept separate from passive Control because participants received an active intervention that may independently improve mobility, balance, or quality of life. |
| Pilates | General Pilates intervention not otherwise specified as mat, reformer, clinical, home-based, online, tele-Pilates, Pilates-TR, or PBCST. | Pilates; Pilates group; Pilates (Pre/Post); Pilates with Music if pooled under the generic Pilates node. | Represents the generic Pilates category when the original trial did not require a more specific modality node or when the analysis intentionally pooled similar Pilates arms. |
| Pilates Training | Structured Pilates exercise program explicitly labelled as Pilates training in the extraction sheet or original report. | Pilates Training; Pilates Training (Pre/Post). | Retained as a separate node because the submitted network and forest plots coded it separately from the generic Pilates node. |
| Pilates-TR | Pilates-based telerehabilitation delivered through a remote rehabilitation format. | Pilates-TR. | Kept as a distinct remote-delivery node because therapist-guided telerehabilitation may differ from in-person, online, and self-directed home programs. |
| Tele-Pilates | Remotely delivered Pilates intervention using tele-exercise or telecommunication platforms. | Tele-Pilates. | Retained separately because the current figures distinguish Tele-Pilates from Pilates-TR and Online Pilates. |
| Online Pilates | Internet-based Pilates program delivered remotely, usually through an online platform. | Online Pilates Group. | Kept as an independent node when trials specifically labelled the intervention as online Pilates and the network coded it separately. |
| Home-Based Pilates | Pilates program performed mainly at home, with self-practice, remote support, or periodic supervision. | Home-Based Pilates; Home-based PBCST if analysed under a home-based Pilates node. | Separated from supervised, tele-, and online nodes because delivery setting and supervision level may affect adherence, dose, and treatment response. |
| Clinical Pilates | Therapeutic Pilates program delivered within a clinical rehabilitation framework, commonly physiotherapist-supervised. | Clinical Pilates. | Kept separate because clinical Pilates is typically individualized and rehabilitation-oriented rather than a general fitness Pilates program. |
| Mat Pilates | Pilates exercises performed on a mat without reformer apparatus. | Mat Pilates. | Separated from Reformer Pilates because equipment use changes exercise mechanics, resistance, and intervention dose. |
| Reformer Pilates | Equipment-based Pilates performed using a reformer apparatus. | Reformer Pilates. | Retained as a modality-specific node because reformer-based training differs from mat-based and generic Pilates interventions. |
| Supervised PBCST | Supervised Pilates-based core stability training focused on core stability, postural control, and functional movement. | Supervised PBCST; Supervised when this label refers to supervised PBCST in the figure output. | Use one harmonized label across the manuscript and figures. If “Supervised” refers to this node, rename it consistently as “Supervised PBCST.” |
| Rebound | Rebound-based exercise or therapy comparator/intervention assessed as a non-Pilates active node. | Rebound; rebound therapy/training if labelled as such in the trial report or network coding file. | Kept separate because it is an active non-Pilates modality and appears as an independent node in the mobility/balance networks. |
| Relaxation | Structured relaxation-based comparator intervention. | Relaxation. | Not merged with passive Control because structured relaxation provides participant contact and may influence patient-reported or functional outcomes. |

Abbreviations: NMA, network meta-analysis; PBCST, Pilates-based core stability training; Pilates-TR, Pilates-based telerehabilitation.
