# Supplementary material for: Comparative effects of pilates-based interventions on functional mobility, balance, fatigue, and quality of life in people with multiple sclerosis: a systematic review and network meta-analysis
Source: BMC Sports Sci Med Rehabil. 2026 Jul 4;18:307. doi: 10.1186/s13102-026-01827-1 (PMC13340119; doi:10.1186/s13102-026-01827-1)
Supplement: Supplementary file 4 — Supplementary Material 4. [file 13102_2026_1827_MOESM4_ESM.docx]

**Supplementary Table 3:** Certainty of evidence

**Patient or population:** Adults (≥18 years) with multiple sclerosis (PwMS), including relapsing-remitting and progressive subtypes; predominantly ambulatory.

**Setting:** Single- and multi-centre randomised controlled trials (RCTs), 2013–2025, conducted in Iran, Turkey, Ireland, Canada, the UK and Israel; supervised, home- and tele-/online-delivered programmes, typically 6–12 weeks.

**Intervention:** Highest-ranked Pilates-based class/agent per outcome by frequentist network meta-analysis (treatments ranked by SUCRA).

**Comparison:** Control (usual care, wait-list, or no exercise) as the network reference.

**Network consistency:** Outcome networks are star-shaped or contain only a few closed loops. Formal incoherence testing (node-splitting; design-by-treatment interaction) was not feasible for outcomes without closed loops (2MWT, 6MWT, MFIS) — acknowledged per PRISMA-NMA 2015 item 17.

**Transitivity:** Assessed qualitatively. Contributing RCTs enrolled PwMS of varying disability (EDSS) and used heterogeneous comparators (wait-list, home exercise, active controls) over 6–12 weeks; for MSQOL-54, possible overlap between Najafi 2023a/b reports — potential transitivity violations acknowledged as study limitations.

| **#** | **Outcomes** | **Anticipated absolute effects* (95% CI)** | | **Relative effect (MD, 95% CI) †** | **No. of participants (studies)** | **Certainty of the evidence (GRADE)** | **Comments** |
| --- | --- | --- | --- | --- | --- | --- | --- |
|  |  | **With Control (reference)** | **With top-ranked intervention (95% CI)** |  |  |  |  |
| **FUNCTIONAL OUTCOMES — BALANCE & MOBILITY** | | | | | | | |
| **1** | **Berg Balance Scale (BBS)** Balance — higher = better Follow-up: 6–12 wk | **Control** (reference) | **8.58 points higher** (7.86 to 9.30 higher) Pilates · SUCRA 0.99 | **MD 8.58** (7.86 to 9.30) | 261 (8 RCTs) | ⊕⊕◯◯  **Low**  *(a,b)* | Pilates ranked 1st; large, statistically significant gain. Closed loop present (Pilates–Rebound–Control). Downgraded for risk of bias (predominantly high-RoB trials) and inconsistency across direct estimates. |
| **2** | **Timed Up-and-Go (TUG)** Mobility — lower (faster) = better Follow-up: 6–12 wk | **Control** (reference) | **5.23 s lower (faster)** (6.39 to 4.06 lower) Pilates · SUCRA 1.00 | **MD −5.23** (−6.39 to −4.06) | 337 (10 RCTs) | ⊕⊕◯◯  **Low**  *(a,b)* | Pilates ranked 1st; significant mobility improvement. Closed loop present (Pilates–Rebound–Control). Downgraded for risk of bias and heterogeneity of direct effects. |
| **WALKING CAPACITY & GAIT SPEED** | | | | | | | |
| **3** | **6-Minute Walk Test (6MWT)** Walking capacity (m) — higher = better Follow-up: 8–12 wk | **Control** (reference) | **50.81 m higher** (0.16 to 101.46 higher) Pilates-TR · SUCRA 0.96 | **MD 50.81** (0.16 to 101.46) | 203 (6 RCTs) | ⊕⊕◯◯  **Low**  *(c,e)* | Pilates-TR ranked 1st; benefit only marginally significant with a very wide CI. Star network — no closed loops, incoherence not testable. Downgraded for serious imprecision. |
| **4** | **2-Minute Walk Test (2MWT)** Walking capacity (m) — higher = better Follow-up: ≈ 8–12 wk | **Control** (reference) | **11.78 m higher** (7.01 lower to 30.57 higher) Supervised PBCST · SUCRA 0.82 | **MD 11.78** (−7.01 to 30.57) | 87 (2 RCTs) | ⊕◯◯◯  **Very low**  *(a,c,e)* | Supervised PBCST ranked 1st but with no significant difference vs Control. Sparse star network of only 2 trials; incoherence not testable. Downgraded for risk of bias and very serious imprecision. |
| **5** | **10-Metre Walk Test (10MWT)** Gait speed (m/s) — higher = better Follow-up: 8–12 wk | **Control** (reference) | **0.18 m/s higher** (0.01 lower to 0.37 higher) Online Pilates · SUCRA 0.96 | **MD 0.18** (−0.01 to 0.37) | 147 (3 RCTs) | ⊕◯◯◯  **Very low**  *(a,c)* | Online Pilates ranked 1st; benefit not statistically significant (CI crosses 0). Closed loop present (Pilates–Relaxation–Control). Downgraded for risk of bias and imprecision. |
| **FATIGUE** | | | | | | | |
| **6** | **Fatigue Severity Scale (FSS)** Fatigue — lower = better Follow-up: 6–12 wk | **Control** (reference) | **2.46 points lower** (3.25 to 1.67 lower) Pilates-TR · SUCRA 0.69 | **MD −2.46 (−3.25 to −1.67)** | 185 (5 trials ) | ⊕⊕⊕◯  **Moderate**  *(c)* | Pilates-TR ranked 1st and was associated with a statistically significant reduction in fatigue severity. However, certainty was downgraded for imprecision because the top-ranked contrast was informed by limited evidence, with a small information size and a single-study contribution to the estimate. |
| **7** | **Modified Fatigue Impact Scale** (MFIS) — lower = better Follow-up: 8–12 wk | **Control** (reference) | **9.50 points lower** (15.93 to 3.07 lower) Home-Based Pilates · SUCRA 0.89 | **MD −9.50** (−15.93 to −3.07) | 165 (4 RCTs) | ⊕⊕◯◯  **Low**  *(a,c)* | Home-Based Pilates ranked 1st; significant reduction in fatigue impact; largest contributing trial (Fleming 2021, n=80). Star network — no closed loops. Downgraded for risk of bias and imprecision. |
| **HEALTH-RELATED QUALITY OF LIFE** | | | | | | | |
| **8** | **MSQOL-54 Physical Health** Quality of life — higher = better Follow-up: 6–12 wk | **Control** (reference) | **17.64 points higher** (5.97 to 29.31 higher) Pilates-TR · SUCRA 0.95 | **MD 17.64** (5.97 to 29.31) | 265 (6 RCTs) | ⊕⊕⊕◯  **Moderate**  *(c)* | Pilates-TR ranked 1st; significant improvement in physical-health QoL (single low-RoB trial). Closed loops present. Downgraded once for imprecision (wide CI). Possible report overlap (Najafi 2023a/b). |
| **9** | **MSQOL-54 Mental Health** Quality of life — higher = better Follow-up: 6–12 wk | **Control** (reference) | **15.86 points higher** (9.20 to 22.52 higher) Active Control (tele-yoga) · SUCRA 0.84 | **MD 15.86** (9.20 to 22.52) | 225 (5 RCTs) | ⊕⊕◯◯  **Low**  *(a,d)* | Top-ranked node is the active comparator tele-yoga (SUCRA 0.84); the highest-ranked Pilates modality was Pilates-TR (MD 16.55, 95% CI 2.24–30.86; SUCRA 0.81; Moderate). Downgraded for risk of bias and indirectness / possible report overlap. |

*** Anticipated absolute effects.** All nine outcomes are continuous (instrument scale scores) synthesised as change-from-baseline mean differences. Following Cochrane/GRADE convention for continuous outcomes, the comparator column shows Control as the reference and the intervention column reports the network mean difference (MD) the intervention group is expected to achieve relative to Control (i.e. “the mean change with the intervention was MD points/seconds/metres higher or lower”). No assumed baseline risk is shown because these outcomes are not binary. **† Relative effect.** A relative measure (risk ratio / odds ratio) is not applicable to continuous outcomes; the comparative effect is the mean difference (MD, 95% CI) taken directly from the network forest plots. SUCRA = surface under the cumulative ranking curve (higher = more likely among the better treatments). PBCST = Pilates-based core stability training; Pilates-TR = Pilates-based telerehabilitation; MD = mean difference.

**Explanatory notes (reasons for downgrading certainty)**

**(a) Risk of bias:** most contributing trials were at high risk of bias or raised some concerns (ROB-2), chiefly absence of participant/personnel blinding (inherent to exercise), with concerns over allocation concealment, attrition and selective reporting. Downgraded one or two levels.

**(b) Inconsistency:** the magnitude of the direct effect varied substantially across contributing trials (a small trial often dominated the pooled estimate).

**(c) Imprecision:** the 95% CI is wide and/or crosses the line of no effect, and the optimal information size is not met (small, often single-study contrasts).

**(d) Indirectness / transitivity:** comparators differ across trials (wait-list, home exercise, active controls) and some estimates draw on potentially overlapping reports (Najafi 2023a/b).

**(e) Incoherence not assessable:** the comparison is informed only by direct evidence within an open, star-shaped network with no closed loops.

**GRADE certainty ratings**

**⊕⊕⊕⊕ High** — further research is very unlikely to change confidence in the estimate.

**⊕⊕⊕◯ Moderate** — further research is likely to have an important impact and may change the estimate.

**⊕⊕◯◯ Low** — further research is very likely to change the estimate.

**⊕◯◯◯ Very low** — the estimate is very uncertain.
