# Supplementary material for: Comparative effects of pilates-based interventions on functional mobility, balance, fatigue, and quality of life in people with multiple sclerosis: a systematic review and network meta-analysis
Source: BMC Sports Sci Med Rehabil. 2026 Jul 4;18:307. doi: 10.1186/s13102-026-01827-1 (PMC13340119; doi:10.1186/s13102-026-01827-1)
Supplement: Supplementary file 5 — Supplementary Material 5. [file 13102_2026_1827_MOESM5_ESM.docx]

**Figures Legend:**

[**Fig.S1** Network diagram of intervention comparisons for Timed Up and Go (TUG) performance](#Fig1)

[**Fig.S2** Network diagram of intervention comparisons for Berg Balance Scale (BBS)](#Fig2)

[**Fig.S3** Network diagram of intervention comparisons for 6-Minute Walk Test (6MWT)](#Fig3)

[**Fig.S4** Network diagram of intervention comparisons for 2-Minute Walk Test (2MWT)](#Fig4)

[**Fig.S5** Network diagram of intervention comparisons for 10 Meter Walk Test (10MWT)](#Fig5)

[**Fig.S6** Network diagram of intervention comparisons for Fatigue Severity Scale (FSS)](#Fig6)

[**Fig.S7** Network diagram of intervention comparisons for Modified Fatigue Impact Scale (MFIS)](#Fig7)

[**Fig.S8** Network diagram of intervention comparisons for MSQOL-54 Physical Health](#Fig8)

[**Fig.S9** Network diagram of intervention comparisons for MSQOL-54 Mental Health](#Fig9)

[**Fig.S10** Forest Plot of MSQOL-54 Physical Health](#Fig10)

[**Fig.S11** Forest Plot of MSQOL-54 Mental Health](#Fig11)

[**Fig.S12** Forest Plot of 2-Minute Walk Test](#Fig12)

[**Fig.S13** Forest Plot of 10-Meter Walk Test](#Fig13)


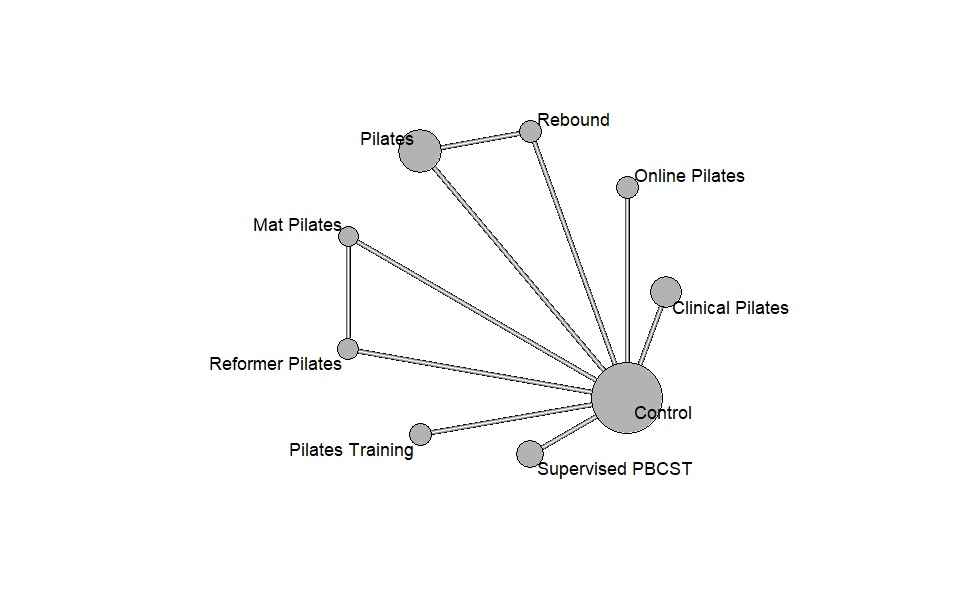


**Fig.S1** Network diagram of intervention comparisons for Timed Up and Go (TUG) performance


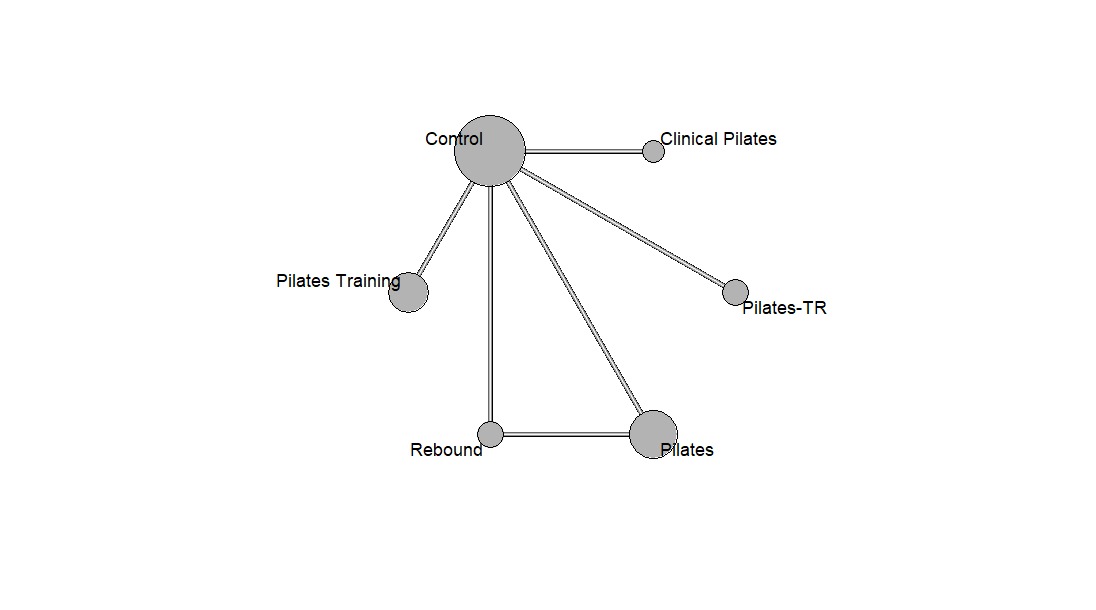


**Fig.S2** Network diagram of intervention comparisons for Berg Balance Scale (BBS)


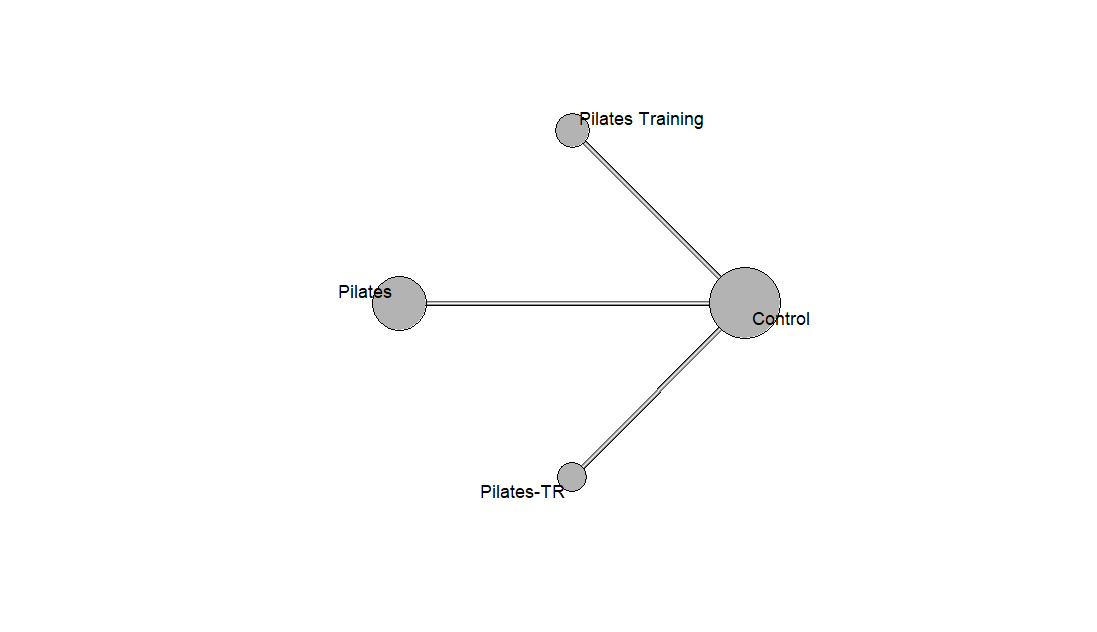


**Fig.S3** Network diagram of intervention comparisons for 6-Minute Walk Test (6MWT)


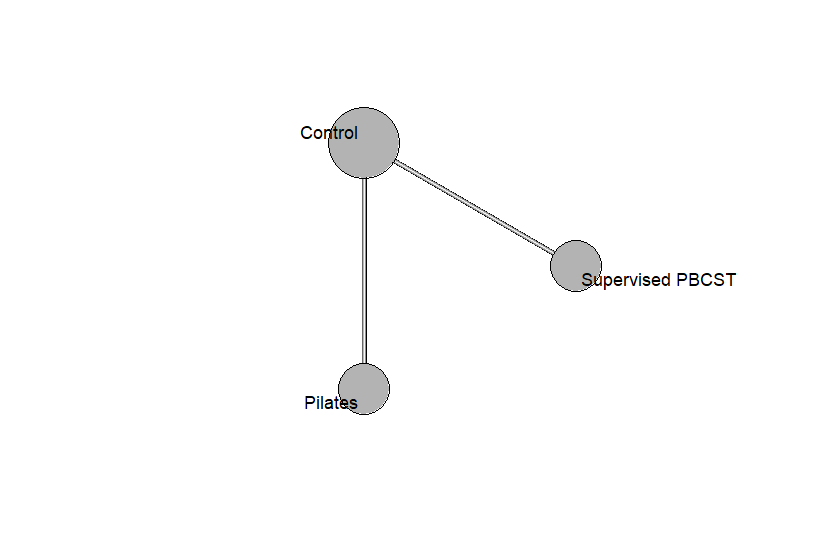


**Fig.S4** Network diagram of intervention comparisons for 2-Minute Walk Test (2MWT)


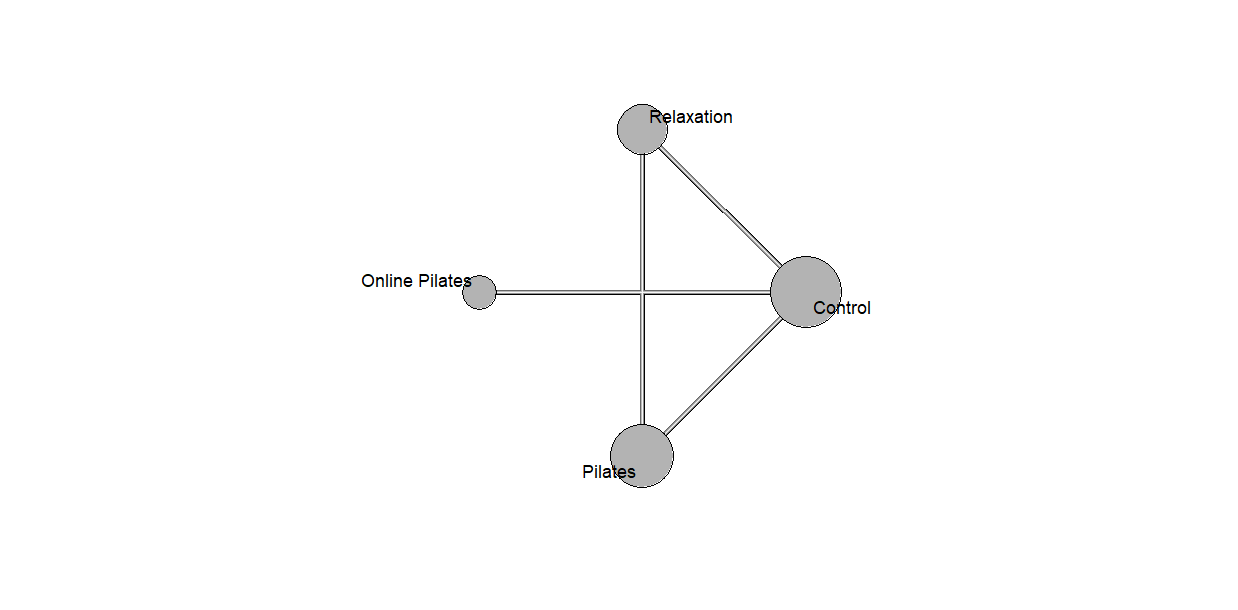


**Fig.S5** Network diagram of intervention comparisons for 10 Meter Walk Test (10MWT)


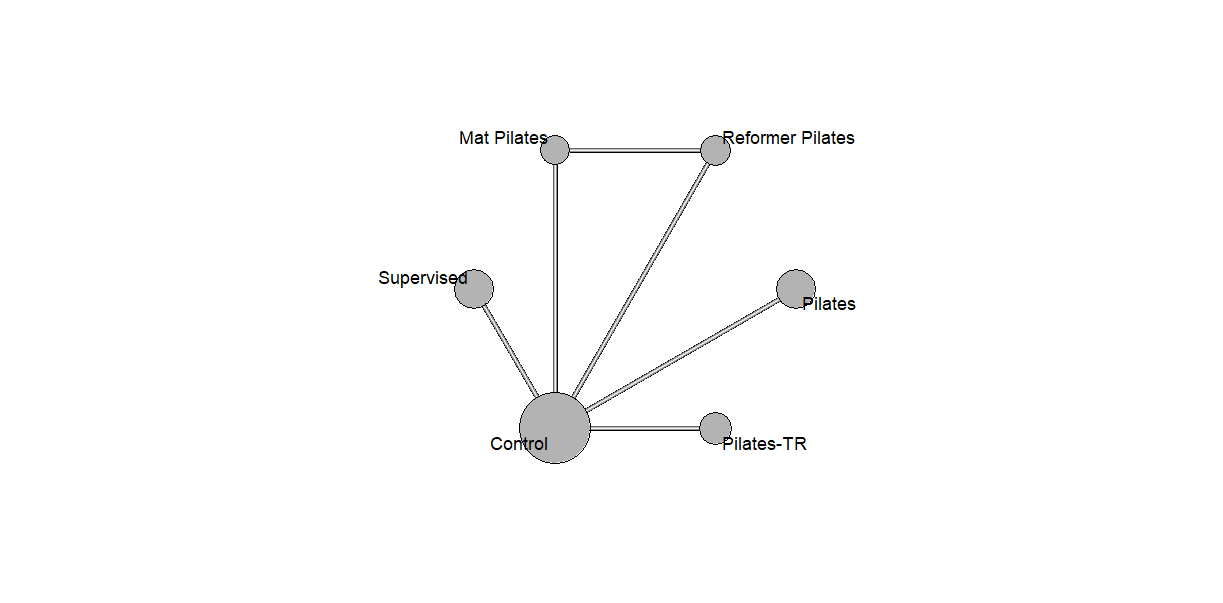


**Fig.S6** Network diagram of intervention comparisons for Fatigue Severity Scale (FSS)


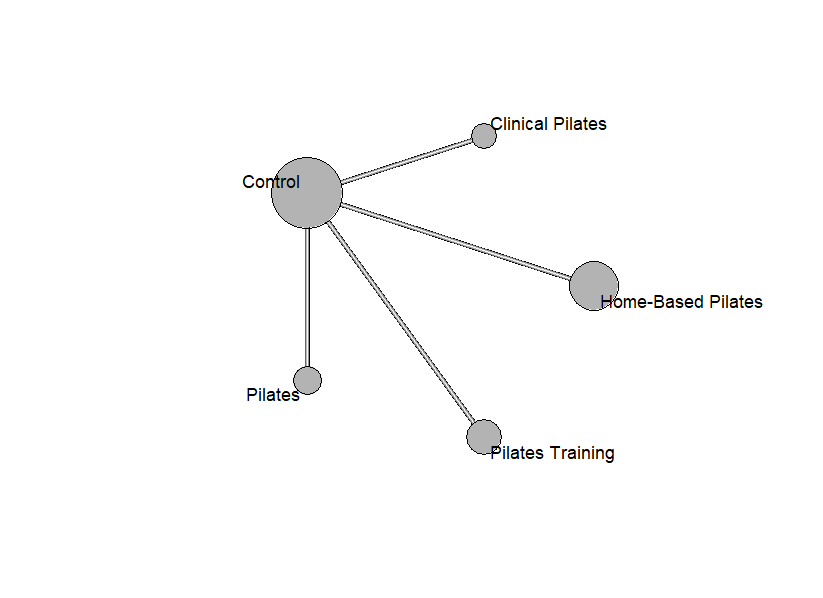


**Fig.S7** Network diagram of intervention comparisons for Modified Fatigue Impact Scale (MFIS)


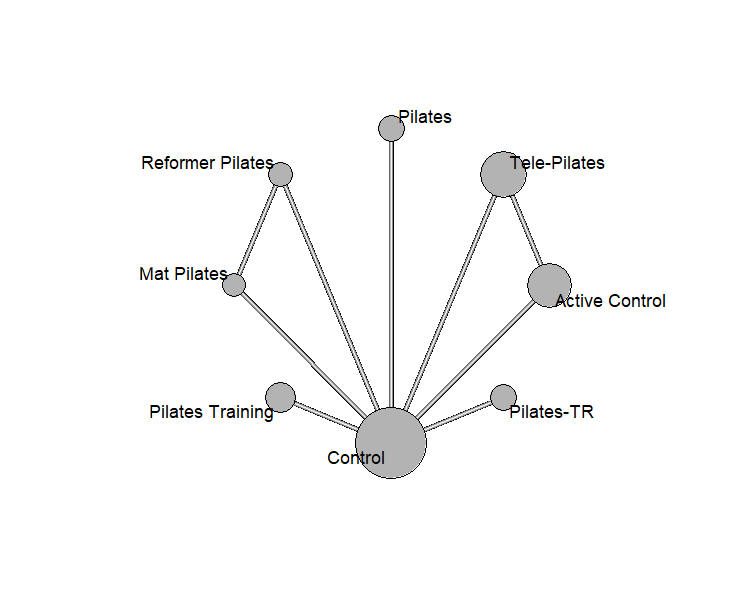


**Fig.S8** Network diagram of intervention comparisons for MSQOL-54 Physical Health


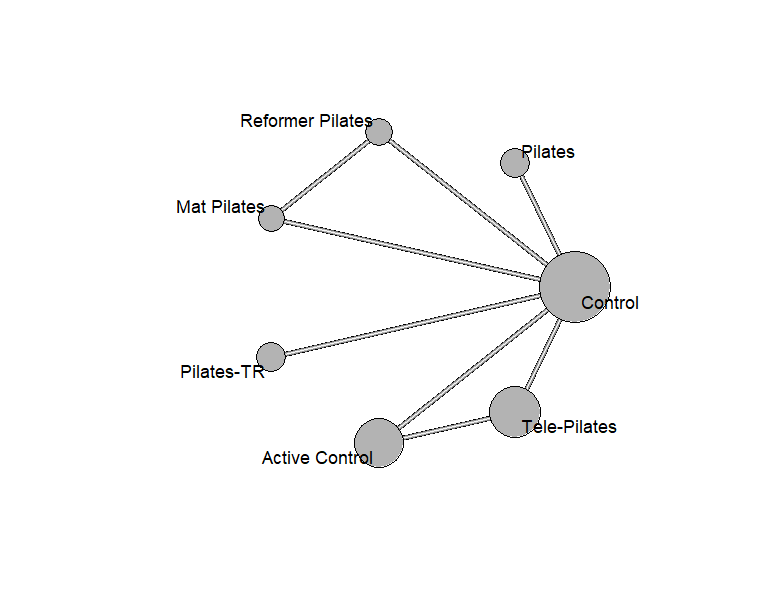


**Fig.S9** Network diagram of intervention comparisons for MSQOL-54 Mental Health


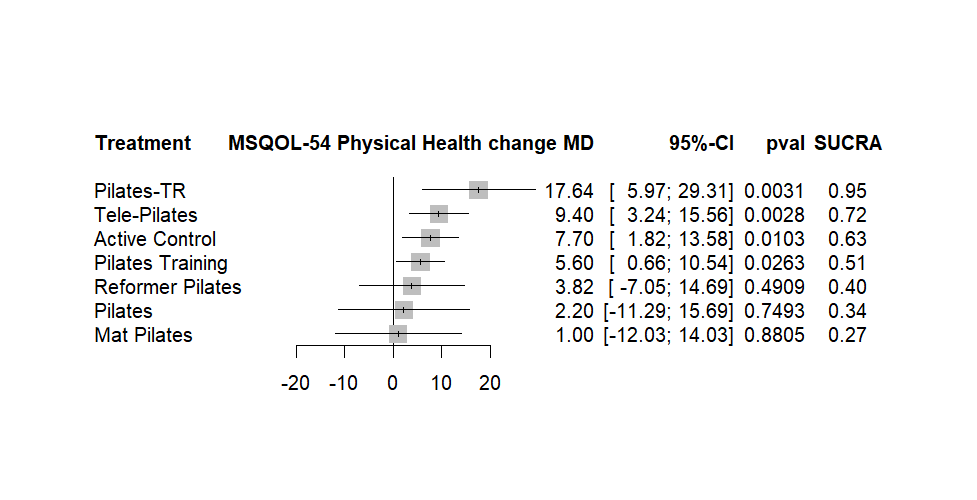


**Fig.S10** Forest Plot of MSQOL-54 Physical Health


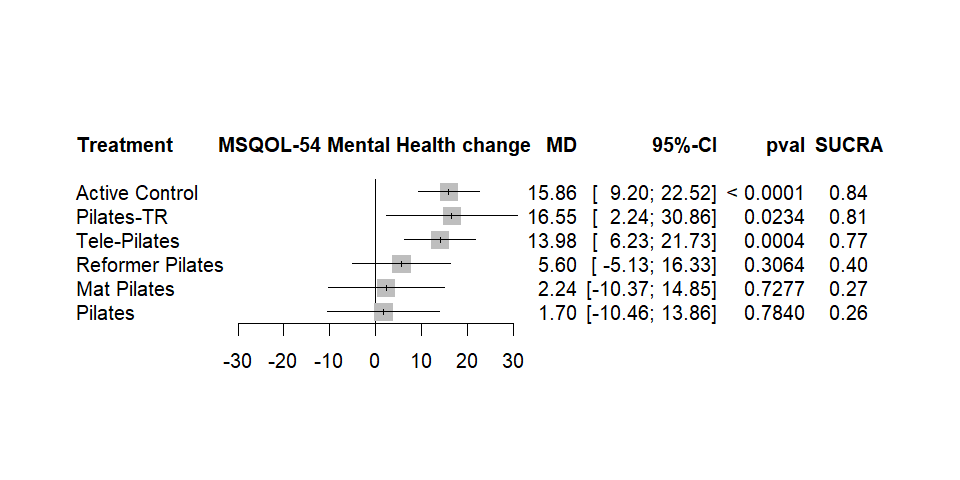


**Fig.S11** Forest Plot of MSQOL-54 Mental Health


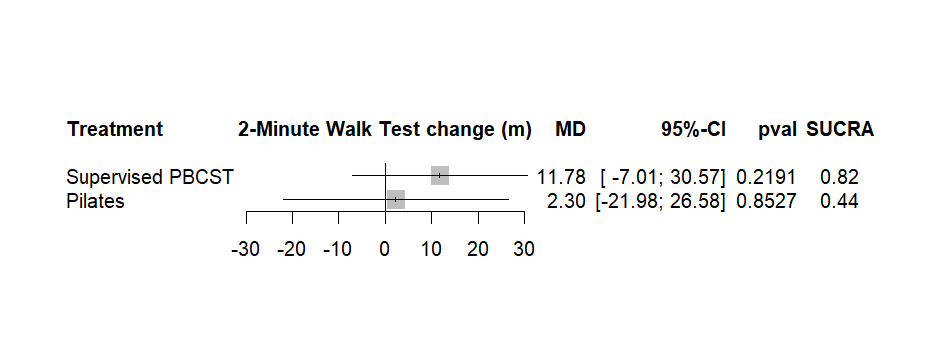


**Fig.S12** Forest Plot of 2-Minute Walk Test


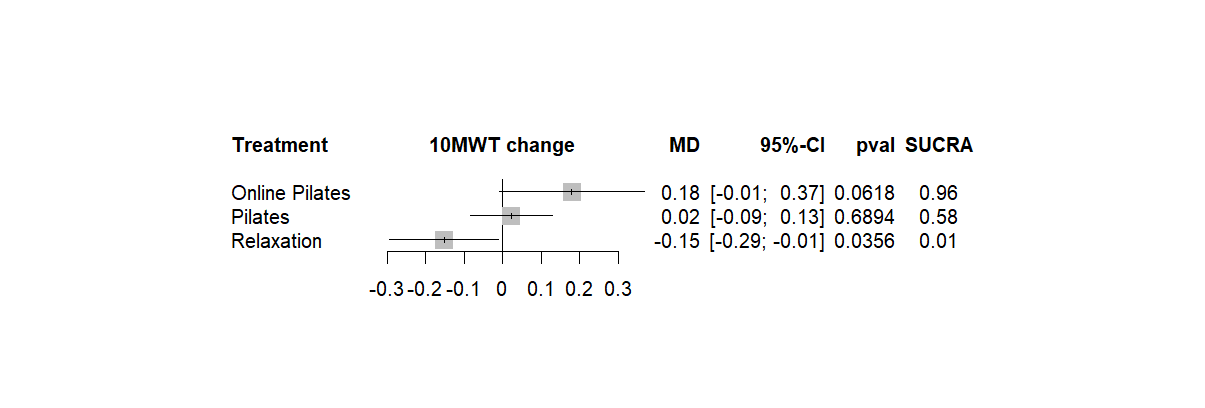


**Fig.S13** Forest Plot of 10-Meter Walk Test
